# Exploring the Origin of Differential Binding Affinities of Human Tubulin Isotypes αβII, αβIII and αβIV for DAMA-Colchicine Using Homology Modelling, Molecular Docking and Molecular Dynamics Simulations

**DOI:** 10.1371/journal.pone.0156048

**Published:** 2016-05-26

**Authors:** Bajarang Vasant Kumbhar, Anubhaw Borogaon, Dulal Panda, Ambarish Kunwar

**Affiliations:** Department of Biosciences and Bioengineering, Indian Institute of Technology Bombay, Powai, Mumbai-400076, Maharashtra, India; Hiroshima Universtiy, JAPAN

## Abstract

Tubulin isotypes are found to play an important role in regulating microtubule dynamics. The isotype composition is also thought to contribute in the development of drug resistance as tubulin isotypes show differential binding affinities for various anti-cancer agents. Tubulin isotypes αβ_II_, αβ_III_ and αβ_IV_ show differential binding affinity for colchicine. However, the origin of differential binding affinity is not well understood at the molecular level. Here, we investigate the origin of differential binding affinity of a colchicine analogue N-deacetyl-N-(2-mercaptoacetyl)-colchicine (DAMA-colchicine) for human αβ_II_, αβ_III_ and αβ_IV_ isotypes, employing sequence analysis, homology modeling, molecular docking, molecular dynamics simulation and MM-GBSA binding free energy calculations. The sequence analysis study shows that the residue compositions are different in the colchicine binding pocket of αβ_II_ and αβ_III_, whereas no such difference is present in αβ_IV_ tubulin isotypes. Further, the molecular docking and molecular dynamics simulations results show that residue differences present at the colchicine binding pocket weaken the bonding interactions and the correct binding of DAMA-colchicine at the interface of αβ_II_ and αβ_III_ tubulin isotypes. Post molecular dynamics simulation analysis suggests that these residue variations affect the structure and dynamics of αβ_II_ and αβ_III_ tubulin isotypes, which in turn affect the binding of DAMA-colchicine. Further, the binding free-energy calculation shows that αβ_IV_ tubulin isotype has the highest binding free-energy and αβ_III_ has the lowest binding free-energy for DAMA-colchicine. The order of binding free-energy for DAMA-colchicine is αβ_IV_ ≃ αβ_II_ >> αβ_III_. Thus, our computational approaches provide an insight into the effect of residue variations on differential binding of αβ_II_, αβ_III_ and αβ_IV_ tubulin isotypes with DAMA-colchicine and may help to design new analogues with higher binding affinities for tubulin isotypes.

## Introduction

**Fig 1:**
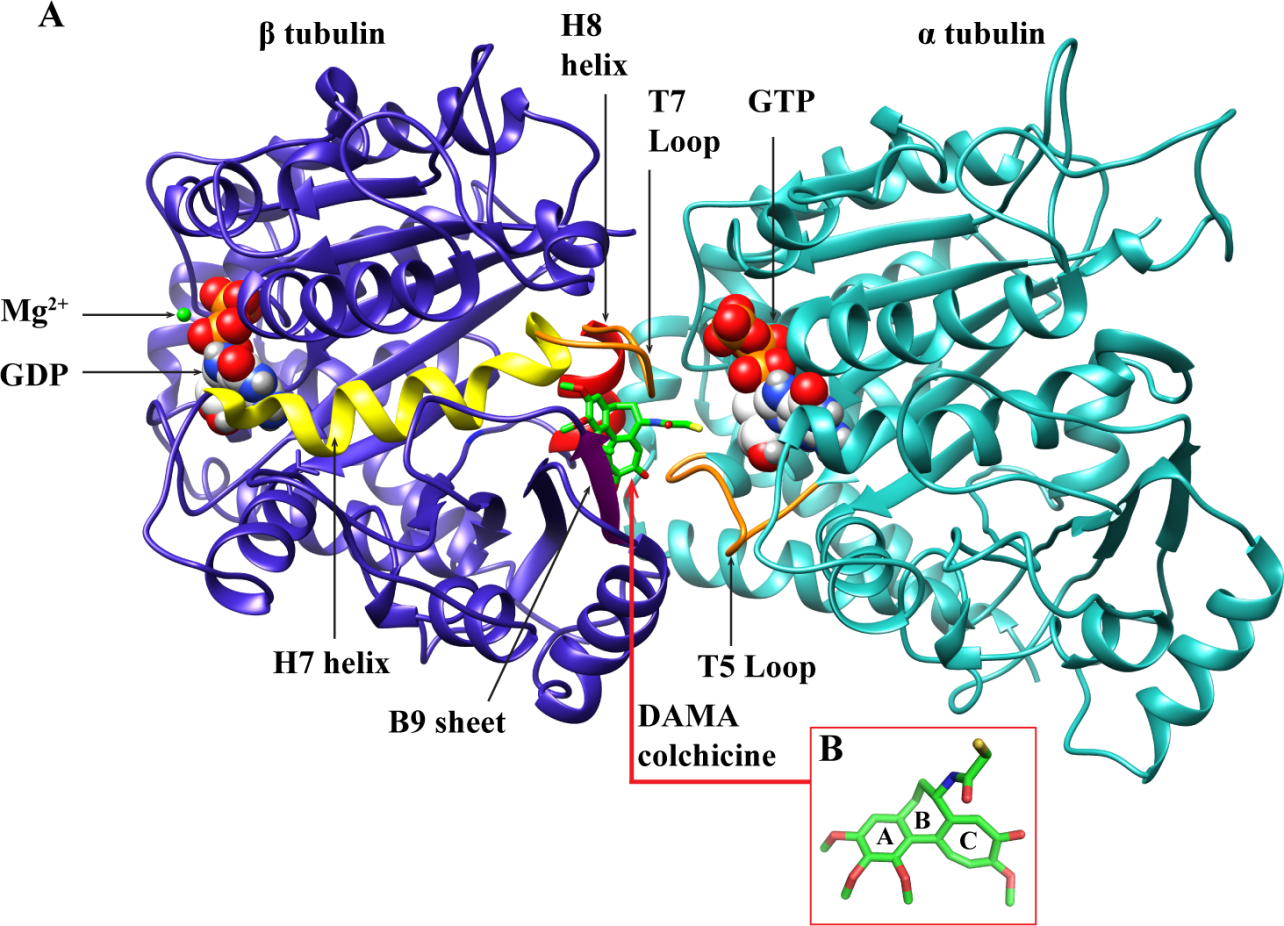
The αβ-tubulin dimer and DAMA-colchicine. (A) The α-tubulin and β-tubulin heterodimer (PDB ID: 1SA0). α-tubulin is shown in green cyan and β-tubulin is shown in tv_blue color. Regions of colchicine binding pocket are highlighted with different colors; the T5 and T7 loop with orange color, the cylindrical H7 and H8 helices with yellow and red color respectively, and B9 sheet is with magenta color. The GTP and GDP are shown using spacefill models. The white, grey, red, blue and golden yellow colors represent carbon, hydrogen, oxygen, nitrogen and phosphorous atoms, respectively. The DAMA-colchicine has been shown using stick model where green, grey, blue, red and yellow colors represent carbon, hydrogen, nitrogen, oxygen and sulphur atoms respectively (B) Structure of DAMA-colchicine: Ring A and C show trimethoxy benzene and methoxytropone ring and seven member B ring join A and C ring with mercaptoacetyl group.

Microtubules (MTs) play crucial roles in various important cellular functions such as cell division, cell motility, transport of vesicles, cell signaling, cell shaping and sensory transduction [1]. They are polymers of a heterodimeric protein, tubulin (Fig 1A). The α-tubulin and β-tubulin are encoded by multiple genes expressed in a tissue specific manner [2–5], such differential expression of tubulin isotype has functional significances [6–8]. Several β-tubulin isotypes are observed in mammals [2–4, 6, 9, 10]. In humans, eight isotypes of β-tubulin have been observed which are expressed differently in different tissues [4, 9, 11]. β_I_ tubulin isotype is the most abundant type and is constitutively expressed, whereas β_III_ expression is restricted to neuronal tissues and testis. Cancerous cells deregulate tissue-specific expression of different isotypes; particularly the overexpression of β_III_ has been associated with aggressive drug resistant cancer cells [12–14]. Mutations in tubulin have also been associated with certain diseases e.g. Polymicrogyria (PMG), Malformation of Cortical Development (MCD) and Congenital Fibrosis of Extraocular Muscle type 3 (CFEOM3) [15–17].

In the present study, we investigate the binding affinity of different β-tubulin isotypes for colchicine analogue N-deacetyl-N-(2-mercaptoacetyl)-colchicine (DAMA-colchicine). Colchicine is the most widely studied anti-mitotic agent to understand the dynamics and function of microtubules. Colchicine is composed of three rings i.e. trimethoxy benzene ring (A ring), methoxytropone ring (C ring), both of which are attached to seven member B ring which has acetamido group at C7 position [18]. In DAMA-colchicine, acetamido group is replaced by mercaptoacetyl group at C7 position (Fig 1B). It has been shown that colchicine binds to tubulin with a very high affinity [19], and induces conformational changes in tubulin [20]. The X-ray crystallography study of αβ-tubulin (PDB ID: 1SA0, resolved at 3.58Å) shows that DAMA-colchicine binds at the interface of αβ-tubulin dimer [21]. α-tubulin and β-tubulin share 40% sequence similarity and each can be divided into the three functional domains; the N-terminal domain (1–205) containing the nucleotide binding region, intermediate domain (206–381), and C-terminal domain (382–450) (Fig 1A).

There have been various studies to understand the biochemical and biophysical properties of interaction of colchicine with tubulin [18, 22, 23], but its effect on various tubulin isotypes has not yet well been understood. The kinetic binding study of tubulin isotypes with colchicine showed that bovine β-tubulin isotype IV has the highest binding affinity, which is followed by β-tubulin isotype II and III respectively. The order of the binding affinities for colchicine is αβ_IV_ > αβ_II_ ≃ αβ_III_ [24]. However, molecular reasons behind the differential binding affinity of these three tubulin isotypes for colchicine is not understood. Similarly, Banerjee et al. [25] studied the binding of desacetamidocolchicine (DAAC) with bovine tubulin isotypes αβ_II_, αβ_III_ and αβ_IV_. DAAC is a colchicine analogue which lacks the acetamidogroup (NH-CO-CH) of B ring [25]. They found that the on-rate was slowest in case of αβ_III_ isotype, and concluded that the colchicine binding domain on the αβ_III_ isotype may differ from that of αβ_II_ and αβ_IV_ isotypes in a small region that might accommodate the B-ring of colchicine. They further concluded that the colchicine binding region is much less flexible in αβ_III_ isotype. Hence, it is important to explore the origin of these differential binding affinities to develop effective analogues against drug-resistant tubulin isotypes.

In the present study, we employed sequence analysis, homology modeling, molecular docking, molecular dynamics simulations and binding free-energy calculations to investigate the differential binding affinity of the three human tubulin isotypes for colchicine analogue DAMA-colchicine.

## Materials and Methods

### Sequence Analysis of Tubulin Isotype

For multiple sequence analysis study of human β-tubulin isotypes, the sequences of β_II_, β_III_ and β_IV_ were downloaded from the UniProt protein sequence database. The sequence of the template (Tubulin 1SA0) [21] was retrieved from the protein database. Since our template is from bovine, the sequences for bovine β-tubulin isotypes (β_II_, β_III_ and β_IV_) were also downloaded from the UniProt protein sequence database for comparison with human β-tubulin isotypes. The multiple sequence alignment of β-tubulin isotypes and template sequence were performed using online multiple sequence analysis tools of EMBL-EBI [26].

### Homology Modeling of Tubulin Isotypes

To find out the mechanism of binding and the binding affinity of DAMA-colchicine with different human β-tubulin isotypes, we constructed 3D models of αβ_II_, αβ_III_ and αβ_IV_ isotypes using homology modeling, since it is known both experimentally [21] and computationally [27] that colchicine binds at the interface of α tubulin and β tubulin. A comparison of human β-tubulin isotypes β_II_ (Uniprot ID: Q13885), β_III_ (Uniprot ID: Q13509) and β_IV_ (Uniprot ID: P04350) with bovine tubulin isotypes β_II_ (Uniprot ID: E1BJB1), β_III_ (Uniprot ID: Q2T9S0) and β_IV_ (Uniprot ID: Q3ZBU7) using multiple sequence alignment, shows that they are identical i.e. 100% similar. We used crystal structure 1SA0.pdb [21] as a template for building 3D homology models of human αβ-tubulin isotypes. 1SA0.pdb is from bovine. Bovine β_II_ tubulin (Uniprot ID: Q6B856) is identical in sequence to human β_II_ tubulin (Uniprot ID: Q9BVA1). This was the reason for choosing 1SA0.pdb as a template to build the 3D model of three tubulin isotypes from human.

We selected chains A and B, containing GTP and Mg^2+^ in α tubulin, and GDP and Mg^2+^ in β tubulin. Other chains C & D, and ligands including stathmin like domain were removed from the crystal structure using PyMol [28]. The missing amino acid residues of α tubulin (amino acid 37 to 47) and β tubulin (amino acid 1, 275–284) were modeled using the MODELLER 9v7 program [29]. The best model was selected on the basis of DOPE (Discrete optimized protein energy) score which is statistical potential optimized for model assessment [29]. The homology models of different tubulin isotypes were then built, using above refined crystal structure of tubulin 1SA0.pdb (Fig 1) as a template, with the help of MODELLER 9v7 [29]. The stereo-chemical quality of template (referred as tubulin 1SA0 hereafter) and different tubulin isotypes were evaluated using PROCHECK [30], Verify-3D [31] and ERRAT [32] to check the reliability of generated models, whose details are provided in Text A in S1 Text.

The energy minimization for 5000 cycles was performed over the tubulin 1SA0 and different tubulin isotypes heterodimers i.e. αβ_II_, αβ_III_, and αβ_IV_, to remove steric clashes using AMBER 12 [33], where initial 200 cycles were done through steepest descent method, followed by remaining cycles through conjugate gradient method. The parameters for GTP, GDP and Mg^2+^ were obtained from AMBER parameter database [34, 35] for minimization. These energy minimized tubulin 1SA0 and αβ tubulin isotype heterodimers were then used for molecular docking of DAMA-colchicine using AutoDock4.2 [36].

### Molecular Docking of DAMA-colchicine and αβ Tubulin Isotypes

To understand the putative binding pocket and the residues involved in the bonding and non-bonding interactions, molecular docking of DAMA-colchicine with tubulin 1SA0 and tubulin isotypes αβ_II_, αβ_III_ and αβ_IV_ were carried out using AutoDock4.2 [36]. The three dimensional co-ordinates of DAMA-colchicine from the crystal structure (PDB ID: 1SA0) were used for molecular docking calculations. Since it is known from earlier studies [37] that colchicine binds at αβ tubulin interface [21], hence the putative binding pocket was defined only at the interface of αβ tubulin using AutoGrid [36]. The grid was chosen with a grid spacing of 0.375Å centered on the selected flexible residues present in the active site of αβ interface. The grid box enclosed the entire colchicine binding site at αβ interface, and provided enough conformational space to the DAMA-colchicine for translation and rotation for achieving the best binding conformation.

The Lamarckian genetic algorithm (LGA) was used for docking studies, the step size of 2Å for translation and 50ଊ of rotation were chosen. The maximum number of energy evaluation was set to 25,00,000. Total 50 runs were performed, and for every independent run a maximum number of 27,000 generations were generated on a single population of 150 individuals. The clusters were then compared on the basis of the cluster size and binding energy calculated by AutoDock4.2 scoring function. The lowest energy docked conformation was selected as the probable stable conformation at the αβ tubulin interface binding site. The DAMA-colchicine docked complex of tubulin 1SA0 as well as DAMA-colchicine docked complex of tubulin isotypes αβ_II_, αβ_III_, and αβ_IV_ were further used for molecular dynamics simulation study.

### Electrostatic Contact Potential Calculation

The electrostatic contact potentials were calculated using PyMol [28] using the lowest energy docked complex of DAMA-colchicine with tubulin 1SA0 as well as αβ_II_, αβ_III_, and αβ_IV_ tubulin isotypes after removing α tubulin. A similar strategy was applied while calculating electrostatic contact potential using molecular dynamics (MD) simulated structure of tubulin 1SA0 as well as tubulin isotypes αβ_II_, αβ_III_, and αβ_IV_.

### Molecular Dynamics (MD) Simulations

Molecular dynamics simulation were performed for DAMA-colchicine docked complexes of tubulin 1SA0 and tubulin isotypes (αβ_II_, αβ_III_ and αβ_IV_) using the sander module of AMBER 12 [33]. The AMBER ff99SB force field was used for tubulin, the parameter for GTP, GDP and Mg^2+^ were obtained from AMBER parameter database [34, 35]. DAMA-colchicine was parameterized using the ‘Antechamber’ module of AMBER 12 similar to earlier studies [18]. The Generalized Born Surface Area (GBSA) implicit solvent model was used to mimic the aqueous environment with parameters described by Tsui [38]. Implicit modeling studies are now widely used to understand the protein-ligand interactions as well as protein folding [18, 39, 40]. The molecular dynamics simulations were performed using the approach used in earlier studies [18]; which are as follows: The energy minimization was performed for 5000 cycles, initial 2000 cycles were done through steepest descent method, followed by 3000 cycles of conjugate gradient method on all atoms of αβ tubulin isotype complex. Next, the system was heated to 300 K for 50ps, shake algorithm was applied with a restraint weight of 2 kcal/mol Å^2^. Heating was followed by the equilibration for 500 ps steps. The final production run was performed for 25 ns for each system i.e. tubulin 1SA0 as well as tubulin isotypes (αβ_II_, αβ_III_, αβ_IV_). The non-bonded cutoff distance was set to 15 Å, and trajectories were propagated at 2fs time step, applying shake to freeze the bonds involving hydrogen atoms. The trajectories were saved after each 0.2ps time interval. The PTRAJ module of AMBER 12 was used for analysis of the post molecular dynamics simulation trajectories. Trajectories were then visualized and analyzed using the Visual Molecular Dynamics, VMD [41] and PyMol [28]. The movements of tubulin are not very smooth due to presence of thermal fluctuations in simulations. VMD can smooth the animated trajectories by averaging over a given number of frames. The animations/movies shown in Movies S1–S4 Movies were obtained by setting the Trajectory Smoothing Window Size for tubulin between 5 and 7, and for DAMA-colchicine between 2 and 4.

### Binding Free Energy Calculations

To further investigate the differential binding affinity of different human tubulin isotypes towards DAMA-colchicine, we calculated the binding free energy using MM-GBSA method, which is implemented in the AMBER 12 software [33]. Binding free energy calculations were performed using the mmpbsa module of AMBER 12 software using pairwise Generalized Born model [31]. The binding free energy were calculated using 10000 frames from last 2ns MD trajectories with an interval of 5 for each system. The free energy of complex, receptor and ligand is calculated and energy is estimated as follows in AMBER 12.

$$\Delta G_{bind,\;solvated}=G_{complex,\;solvated}-G_{receptor,\;solvated}-G_{ligand,\;solvated}$$ (1)

The binding free energies, Δ*G*_*solvated*_, is calculated by using following equation using the MM-GBSA method.

$$\Delta G_{solvated}=E_{gas}+E_{sol}-T\Delta S_{solute}$$ (2)

$$E_{gas}=E_{\mathrm{int}}+E_{ele}+E_{vdw}$$ (3)

$$\text{Where},\;E_{\mathrm{int}}=E_{bond}+E_{angle}+E_{torsion}$$ (4)

Where, *E*_*gas*_ is the gas-phase energies are often molecular mechanical energies from force field. *E*_int_ is internal energy which consists of *E*_*bond*_, *E*_*angle*_ and *E*_*torsion*_ which are the bond, angle and torsion energies respectively. The *E*_*ele*_ and *E*_*vdw*_ are the electrostatic and van der Waals interactions energies. The *E*_*sol*_ is the solvation energy and is given by

$$E_{sol}=E_{polar}+E_{non-pol}$$ (5)

$$E_{nonpol}=\gamma SASA$$ (6)

The *E*_*sol*_ is the solvation energy contributions that can be decomposed into the polar and non-polar energy. The polar solvation contribution is calculated by solving the Generalized Born (GB) equation; whereas non polar solvation is estimated by the solvent accessible surface area (SASA), using a water probe radius of 1.4 Å. The surface tension constant (*γ*) was set to 0.0072 kcal/mol A^-2^. The entropy contribution was omitted in this study [42, 43] as entropy calculations are computationally expensive. Thus our binding free energy calculations are similar to earlier studies on tubulin and anticancer drugs [11, 44, 45], and *Streptococcus suis* R61 and cefuroxime drug [46]. The binding free energy calculated will be lower if the entropy contribution is included. Here, we can avoid the need to explicitly calculate the entropy for comparing the relative trend of binding free energies of different isotypes as they are related systems (there is a difference of few residues among them) with respect to experimentally measured relative trend of binding free energy of different isotypes. Thus, one can assume that the solute entropy will be the roughly same for each of these systems which are being compared, and would change the binding free energy by roughly same amount. In mmpbsa module, bonded energy terms (*E*_int_) are not printed for any one-trajectory simulation by default. They are computed and their differences are calculated. However, they are not shown (nor included in the total) in output of mmpbsa module unless specifically asked for because they should cancel completely.

## Results and Discussion

### Sequence Analysis of αβ Tubulin Isotypes

**Fig 2:**
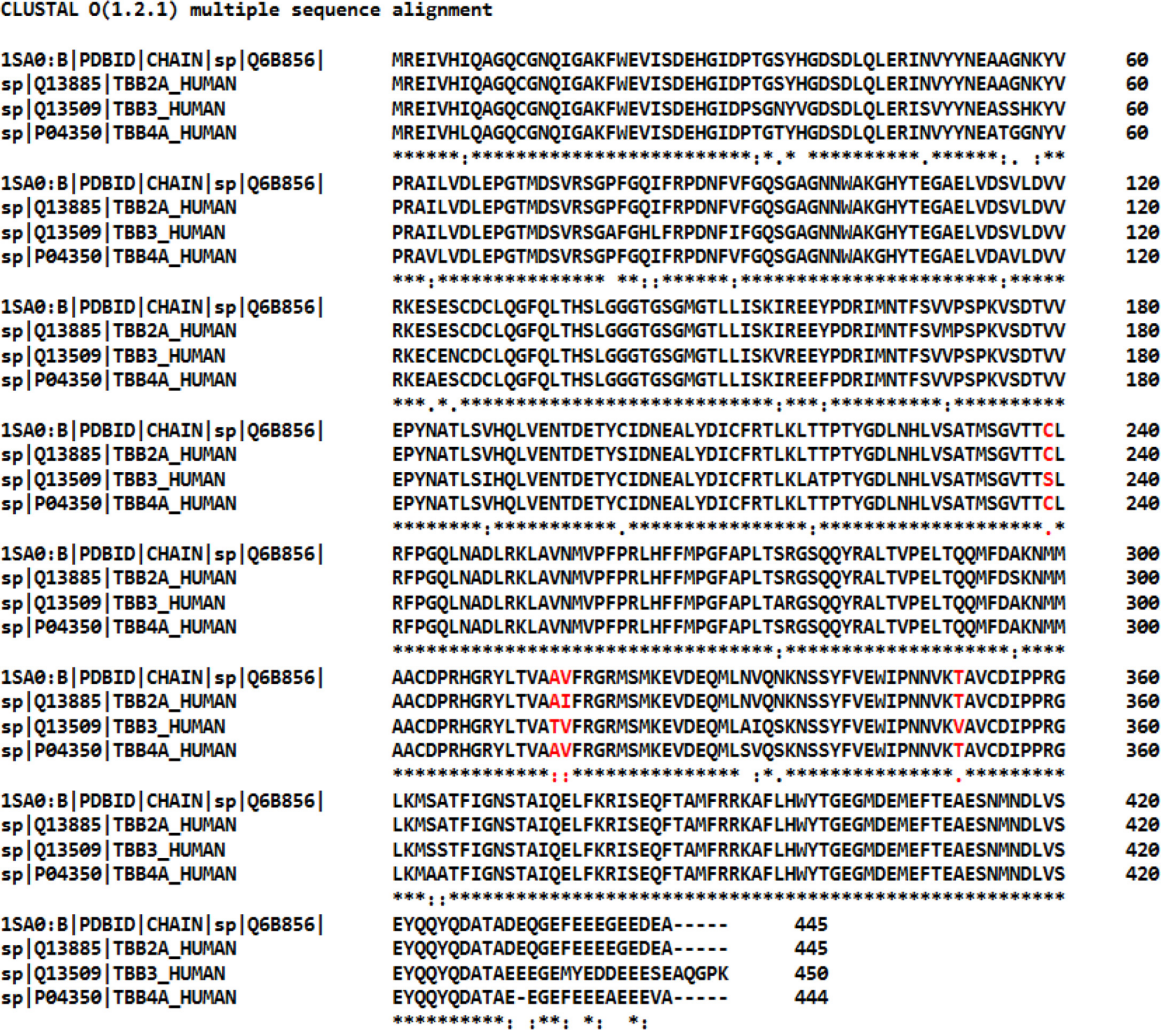
Multiple sequence analysis of tubulin 1SA0 and human β-tubulin isotypes. The isotype β_III_ shows change of Ala315 to Thr, Cys239 to Ser, and Thr351-to Val, Isotype β_II_ change of Val316 to Ile at the colchicine binding pocket. Region of changed residues at colchicine binding pocket are shown in red color.

The multiple sequence alignment of bovine β_II_ tubulin and human β-tubulin isotypes were performed using online multiple sequence analysis tool of EMBL-EBI [26]. The sequence analysis study shows differences in residue composition at different locations in β_II_, β_III_ and β_IV_ isotypes. The analysis of colchicine binding pocket of β_III_ isotype shows changes of three residues i.e. Cys239 to Ser, Ala315 to Thr, and Thr351 to Val (Fig 2), whereas β_II_ isotype contains change of only a single residue i.e. Val316 to Ile at the colchicine binding site. There is no residue change observed in isotype β_IV_ at the colchicine binding pocket (Fig 2). Such differences in the colchicine binding site residues among the isotype β_II_ and β_III_ might contribute in the differential binding of DAMA-colchicine, as observed previously for colchicine [24]. Hence, we performed homology modeling of these tubulin isotypes, followed by docking of DAMA-colchicine, molecular dynamics simulations and binding energy calculations, to understand the effect of change of residue composition observed in different isotypes on the binding of DAMA-colchicine.

### Molecular Docking of αβ Tubulin Isotypes with DAMA-colchicine

**Fig 3:**
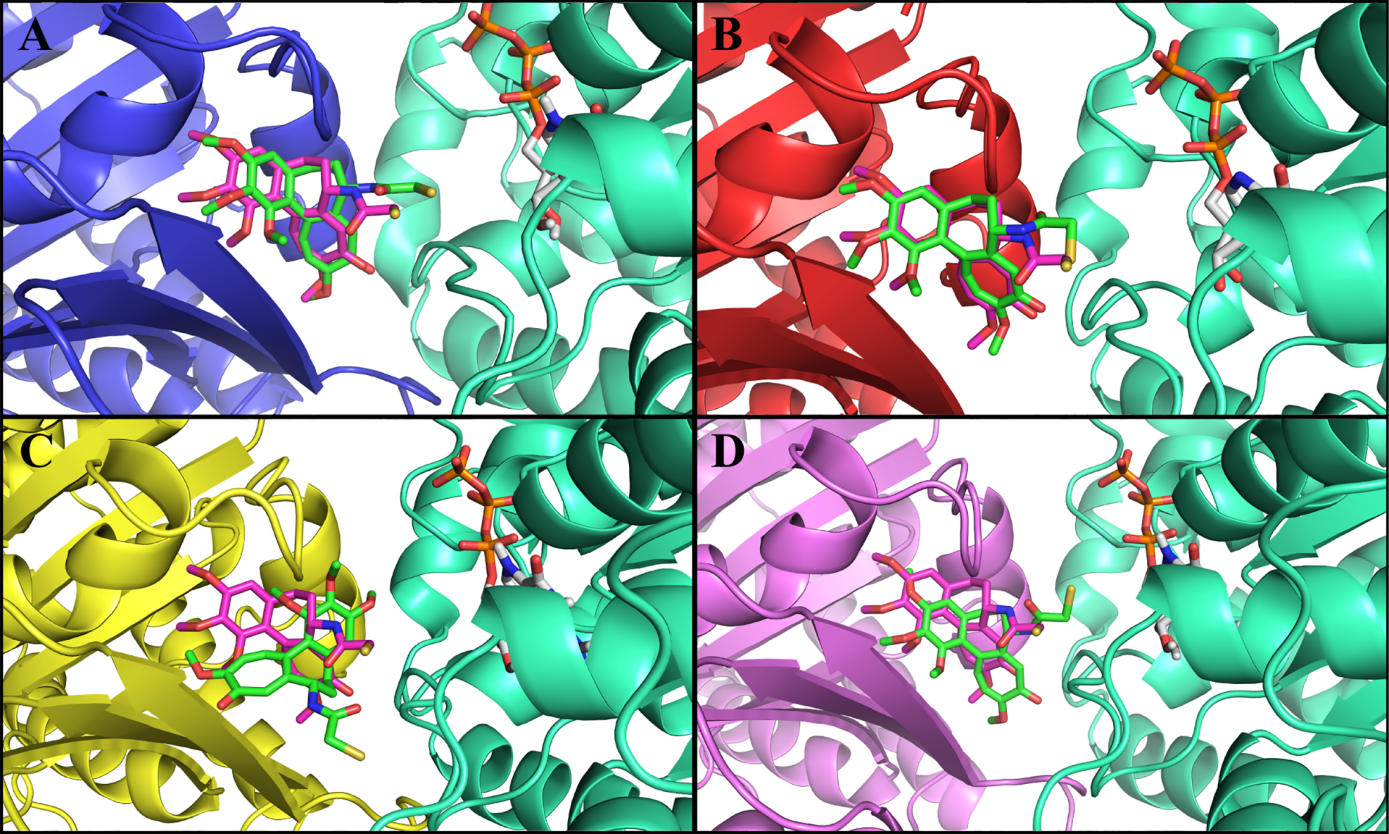
Comparison of crystal structure and docked conformation of DAMA-colchicine in tubulin 1SA0 and human αβ-tubulin isotypes. Color scheme for α-tubulin is green_cyan and β-tubulin is tv_blue for tubulin 1SA0 tv_red for isotype β_II_, tv_yellow for isotype β_III_ and violet for isotype β_IV_. Crystal structure of DAMA-colchicine is shown in magenta color, while DAMA-colchicine after docking is shown in green color. The oxygen, nitrogen and sulphur atoms of DAMA-colchicine are shown in red, blue and pale yellow color, respectively. The DAMA-colchicine prefers the αβ tubulin interface in tubulin 1SA0 and tubulin isotypes. **(A)** Tubulin 1SA0-DAMA-colchicine complex. **(B)** αβ_II_ tubulin isotype-DAMA-colchicine complex **(C)** αβ_III_ tubulin isotype-DAMA-colchicine complex **(D)** αβIV tubulin isotype-DAMA-colchicine complex.

First, we performed a control docking of tubulin 1SA0 with DAMA-colchicine using AutoDock4.2. The binding energy of the minimum energy docked conformation was found to be -10.70 kcal/mol (Table 1). DAMA-colchicine prefers α-tubulin and β-tubulin interface in tubulin 1SA0 (Fig 3A) similar to the crystal structure [21]. The root mean square deviation (RMSD) between the ‘predicted’ and ‘crystallographically’ determined binding modes of DAMA-colchicine was found to be 1.2 Å (Table 1). The RMSD in the range of 2–3 Å indicates appropriate docking [27, 36]. The analysis of binding site residues lying within 4 Å distance indicated that DAMA-colchicine is surrounded by both hydrophobic and hydrophilic residues (Table 2), and stabilized by hydrogen bonding interactions with residues Cys-239 (2.20 Å), Lys-350 (2.31 Å) and Val-181 (1.91 Å) given in Table 1. Cys-239 interacts with the trimethoxy benzene group and Lys-350 and Val-181 interact with the methoxytropone ring of DAMA-colchicine. These hydrogen bonding interactions are shown in.

**Table 1:** RMSD of docked DAMA-colchicine relative to crystal structure, binding energy and hydrogen bonding interactions in tubulin 1SA0, and human αβ_II_ αβ_III_ and αβ_IV_ tubulin isotypes.

Protein system	RMSD of DAMA-colchicine (Å)	Binding energy^a^ (kcal/mol)	Hydrogen bonding interactions			Figure reference
			Atoms involved	Distance (Å)	Angle (Degree)	
**Tubulin 1SA0**	1.2	-10.70	Cys-239-HG….O3-COL	2.20	151.55	3A, Fig A in S5 Fig
			Lys-350-HG….O5-COL	2.31	137.54	
			Val-181-HN….O5-COL	1.91	153.69	
**αβ**_**II**_ **isotype**	2.4	-10.45	Lys-350-HZ3….O5-COL	2.07	103.58	3B, Fig B in S5 Fig
			Cys-239-HG….O3-COL	2.05	133.18	
			Asn-256-HD2…O4-COL	2.15	109.70	
**αβ**_**III**_ **isotype**	5.2	-6.58	Lys-350-HZ3….S1-COL	2.65	113.10	3C, Fig C in S5 Fig
			Asn-101-2HD…O3-COL	2.20	151.58	
**αβ**_**IV**_ **isotype**	2.1	-10.74	Lys-350-HZ1….O5-COL	2.25	175.39	3D, Fig D in S5 Fig
			Cys-239-HG….O3-COL	2.08	160.26	

^a^Binding energy values are obtained from the lowest energy DAMA-colchicine docked complex.

**Table 2:** Residues present around the 4 Å distances of DAMA-colchicine in tubulin 1SA0, and human αβ tubulin isotypes after docking.

Protein	Residues around 4 Å distance of DAMA-colchicine
**Tubulin 1SA0**	**β-tubulin residues:** Cys-239 Leu-246, Ala-248, Lys-252, Leu-253, Asn-256, Met-257, Thr-312, Val-313, Val-316, Asn-348, Lys-350, Ala-352. **α-tubulin residues:** Asn-101, Ser-178, Thr-179, Ala-180, Val-181.
**αβ**_**II**_ **isotype**	**β-tubulin residues:** Val-236, Cys-239, Leu-246, Ala-248, Lys-252, Leu-253, Asn-256, Met-257, Val-313, Ala-314, Val-316, Lys-350, Ala-352, Ile-376. **α-tubulin residues:** Asn-101, Ser-178, Thr-179, Ala-180, Val-181.
**αβ**_**III**_ **isotype**	**β-tubulin residues:** Leu-246, Ala-248, Lys-252, Asn-256, Ala-314, Ala-315, Val-316, Lys-350, Thr-351, Ala-352. **α-tubulin residues:** Asn-101, Pro-175, Ser-178, Thr-179, Ala-180.
**αβ**_**IV**_ **isotype**	**β-tubulin residues:** Cys-239, Leu-246, Ala-248, Lys-252, Leu-253, Asn-256, Val-313, Ala-314, Als-315, Val-316, Asn-346, Asn-348, Val-349, Lys-350, Thr-351, Als-352. **α-tubulin residues:** Asn-101, Thr-179, Ala-180, Val-181.

The molecular docking of DAMA-colchicine on different human tubulin isotypes were performed using the same protocol as used for the control docking of tubulin 1SA0. For docking analysis, we selected the lowest energy docked conformation of DAMA-colchicine with αβ_II_, αβ_III_ and αβ_IV_ tubulin isotypes. In tubulin isotypes, DAMA-colchicine prefers αβ-tubulin interface similar to tubulin 1SA0 (Fig 3A) and crystal structure [21]. The RMSD between ‘predicted’ and ‘crystal structure’ determined binding modes of DAMA-colchicine for αβ_II_ isotype (Fig 3B), αβ_III_ isotype (Fig 3C) and for αβ_IV_ isotype (Fig 3D) were 2.4 Å, 5.2 Å and 2.1 Å, respectively (Table 1). In the αβ_III_ isotype, DAMA-colchicine prefers a different orientation at the interface. The higher RMSD value for αβ_III_ isotype suggests that the changed residue composition (Ser-239, Thr-315, and Val-351) at the colchicine binding pocket might affect the binding.

The analysis of docking complex of αβ_II_ tubulin isotype and DAMA-colchicine (Fig 3B) shows that the DAMA-colchicine binding pocket of αβ_II_ tubulin isotype is amphipathic in nature within 4 Å distance. The residues present around the 4Å distance of DAMA-colchicine are listed in Table 2. The binding energy of DAMA-colchicine was estimated to be -10.45 kcal/mol (Table 1). The analysis of docked complex of αβ_II_ tubulin-DAMA-colchicine shows that DAMA-colchicine forms hydrogen bonding with residue Lys-350 (2.07 Å), Cys-239 (2.05 Å) and Asn-256 (2.15 Å) (Table 1). Here, Lys-350 interacts with the methoxytropone ring, Asn-258 with mercaptoacetyl group and Cys-239 with trimethoxy benzene ring of DAMA-colchicine (Fig B in S5 Fig). In αβ_II_ tubulin-DAMA-colchicine complex, DAMA-colchicine shows similar hydrogen bonding as found in tubulin 1SA0, along with this it also shows additional bonding with Asn-256 (Table 1). The hydrogen bonding interactions for docking complex of αβ_II_ tubulin isotype and DAMA-colchicine are shown in Fig B in S5 Fig.

The analysis of docked complex of αβ_III_ tubulin isotype and DAMA-colchicine (Fig 3C) shows that the DAMA-colchicine prefers a different orientation at the αβ_III_ tubulin interface (Fig 3C) as compared to tubulin 1SA0 (Fig 3A) and αβ_II_ (Fig 3B). The residues present around the 4Å distance of DAMA-colchicine are listed in Table 2. The binding energy of DAMA-colchicine for αβ_III_ was estimated to be -6.58 kcal/mol by docking analysis (Table 1). DAMA-colchicine forms hydrogen bonding (Table 1) with residues Lys-350 (2.65Å) of β-tubulin and Asn-101(2.20Å) of α-tubulin. Due to an altered orientation of DAMA-colchicine, Lys-350 interacts with mercaptoacetyl group and Asn-101 with trimethoxy benzene ring of DAMA-colchicine (Fig C in S5 Fig).

An analysis of docking complex of αβ_IV_ tubulin isotype and DAMA-colchicine (Fig 3D) shows that, DAMA-colchicine also prefers αβ_IV_ tubulin interface similar to tubulin 1SA0 (Fig 3A) and αβ_II_ tubulin isotype (Fig 3B). The residues around the 4Å distance of docked DAMA-colchicine are shown in Table 2, DAMA-colchicine is surrounded by more residues from the β-tubulin chain and very few residues of α-tubulin. The lowest binding energy of DAMA-colchicine was estimated to be -10.74 kcal/mol by docking analysis Table 1. DAMA-colchicine forms hydrogen bonding interactions with Lys-350 (2.25Å) and Cys-239 (2.08Å). Lys-350 interacts with the oxygen atom of methoxytropone ring and Cys-239 with trimethoxy benzene ring at the binding site. These interactions are shown in Fig D in S5 Fig.

It has been observed that, in αβ_II_ and αβ_IV_ tubulin isotype Lys-350 forms hydrogen bonding interactions with methoxytropone ring and Cys-239 with trimethoxy benzene ring of DAMA-colchicine. Whereas, in the altered orientation of DAMA-colchicine at the interface of αβ_III_ isotype, Lys-350 forms hydrogen bonding with mercaptoacetyl group of B ring. The changes in residue composition at the binding pocket produces a profound effect on the binding of DAMA-colchicine. Hence the residues such as Cys-239 and Lys-350 could play a crucial role in binding of DAMA-colchicine.

Molecular Docking studies show that binding energies of tubulin isotypes for DAMA-colchicine (Table 1) are in the order αβ_IV_ ≃ αβ_II_ >> αβ_III_, whereas experimentally measured binding energies of tubulin isotypes for colchicine are in the order αβ_IV_ > αβ_II_ ≃ αβ_III_ for these three isotypes in bovine (Text B in S1 Text) [2].

We calculated electrostatic contact potential over the β tubulin-DAMA-colchicine complex using PyMol [28], since changes in residue composition observed in β-tubulin isotypes show a pronounced effect on the binding of DAMA-colchicine (S6 Fig). The electrostatic contact potential of tubulin 1SA0 (Fig A in S6 Fig), αβ_II_ and αβ_IV_ tubulin isotypes (Fig B in S6 Fig and Fig D in S6 Fig) shows that the DAMA-colchicine is located inside the binding pocket of β-tubulin. While in case of αβ_III_ tubulin isotype, DAMA-colchicine is partially buried inside the binding pocket (Fig C in S6 Fig). Electrostatic potential calculations further confirm that the DAMA-colchicine takes different orientations at the αβ interface of αβ_III_ tubulin isotype. Therefore, to understand the detailed molecular mechanism of differential binding of tubulin isotypes for DAMA-colchicine, we performed molecular dynamics simulations and binding free energy calculations.

### Molecular Dynamics (MD) Simulation of Tubulin heterodimers-DAMA-colchicine Complexes

**Fig 4:**
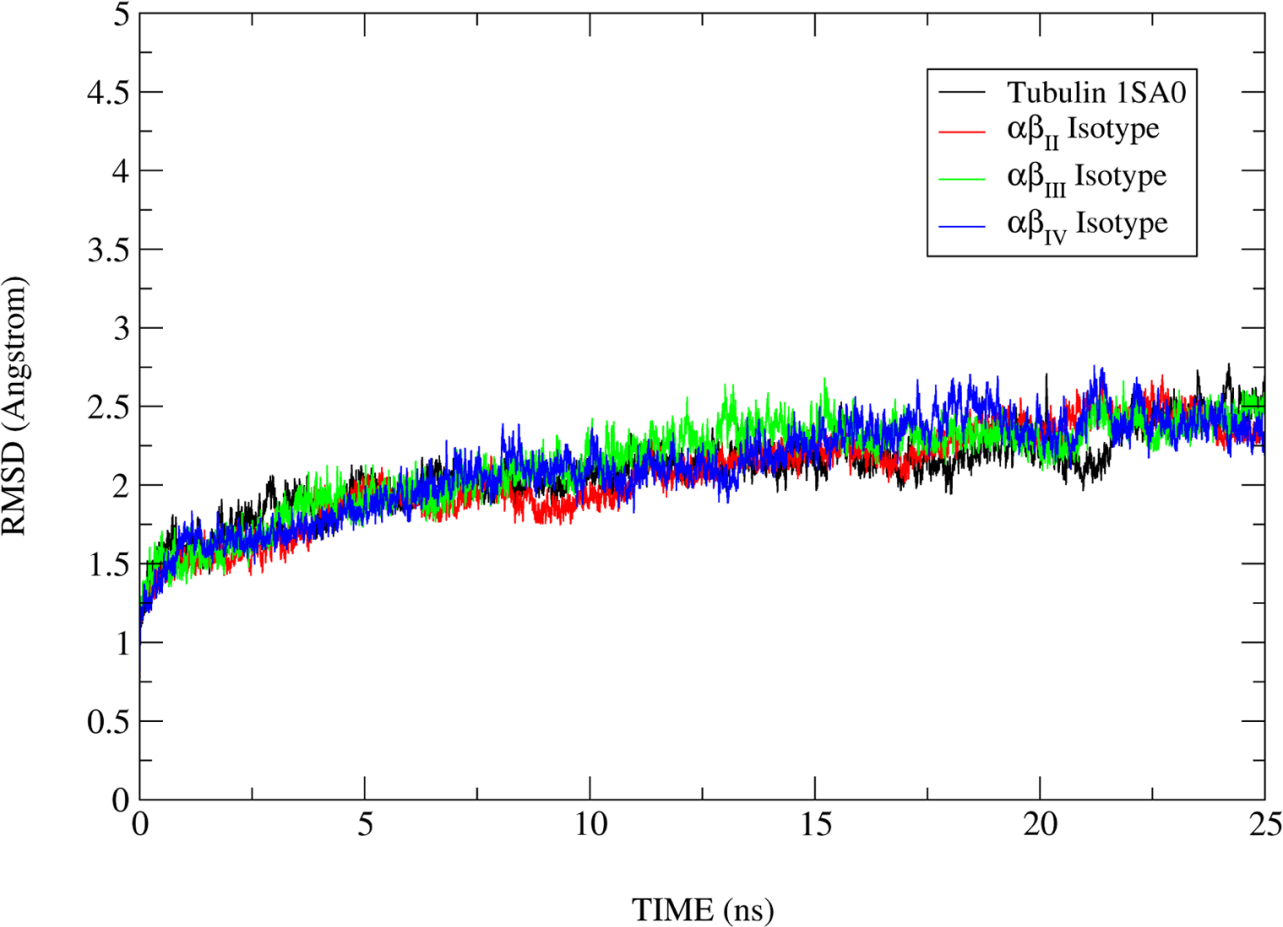
Root mean square deviations (RMSD) corresponding to tubulin 1SA0 and αβ-tubulin isotypes. Root mean square deviations (RMSD) correspond to tubulin 1SA0 (black colour), αβ_II_ (red colour)), αβ_III_ (green colour) and αβ_IV_ (blue colour) of tubulin isotypes for 25ns MD simulations. RMSD was calculated for αβ-tubulin isotypes excluding the C-terminal region starting from amino acid Ala-428. The RMSD analysis suggests that tubulin 1SA0, αβ_II,_ αβ_III_ and αβ_IV_ deviate to quite some extent from their starting conformations and reached their equilibrium conformations after 15ns, and then retained their stability with fluctuations between 2.2–2.7Å.

Molecular dynamics simulations were performed using the lowest energy DAMA-colchicine docked complex of tubulin heterodimers as starting structures (Fig 3A–3D). The primary analysis was made by looking at the MD simulation stability and analysis of protein structure. To check the stability of molecular dynamics simulation, the RMSD of the C_α_ backbone atom of a production dynamics for tubulin 1SA0 and tubulin isotype heterodimers were calculated and are shown in Fig 4. Structurally, the human β-tubulin isotypes differ in composition due to last 15 to 20 C-terminal amino acids (Fig 2), which are the putative binding sites for many microtubule associated proteins (MAPs). Our template structure tubulin 1SA0 doesn’t have C-terminal region in its crystal structure [21]. Therefore, the RMSD of αβ tubulin isotypes considering the last 15 to 20 C-terminal amino acid region of β-tubulin shows an increased RMSD. Hence, we calculated the RMSD excluding the C-terminal region starting from amino acid Ala-428. The RMSD analysis shown in Fig 4 suggests that tubulin 1SA0, αβ_II,_ αβ_III_ and αβ_IV_ deviate to quite some extent from their starting conformations and reached their equilibrium conformations after 15ns, and then retained their stability with fluctuations between 2.2–2.7Å. To understand the compactness of tubulin structure, we calculated the radius of gyration (R_g_) and root mean square of fluctuations (RMSF) of tubulin 1SA0 and different tubulin isotypes.

It is seen that the T7 loop starts moving backwards and DAMA-colchicine is adapted inside the β-tubulin binding site (S1, S2 and S4 Movies) tubulin 1SA0 and tubulin isotypes αβ_II_ and αβ_IV_. Whereas, in the β_III_ tubulin isotype DAMA-colchicine is expelled from the binding pocket. It starts moving out to the surface of the interface (S3 Movie) and closure of the binding pocket starts thereafter. In case of αβ_III_ tubulin isotype, T7 loop starts to move forward while the H7-H8 helix shows less fluctuation as compared to tubulin 1SA0 and other isotypes.

The radius of gyration (R_g_) value indicates the level of compactness of a protein and it has been applied to obtain an insight into the stability of a system during MD simulation [47]. Since change in residue composition are present only on β monomers, the radius of gyration of only β monomer of tubulin 1SA0 and tubulin isotypes were calculated and are shown in S7 Fig. The radius of gyration results in S7 Fig shows the compactness and stable behavior of β-tubulin structures, during molecular dynamics simulations, for tubulin 1SA0 and three β-tubulin isotypes. To understand the effect of changes in residue composition in β-tubulin isotypes, the root mean square fluctuations were calculated and are discussed in detail below.

### Analysis of Root Mean Square Fluctuations (RMSF)

**Fig 5:**
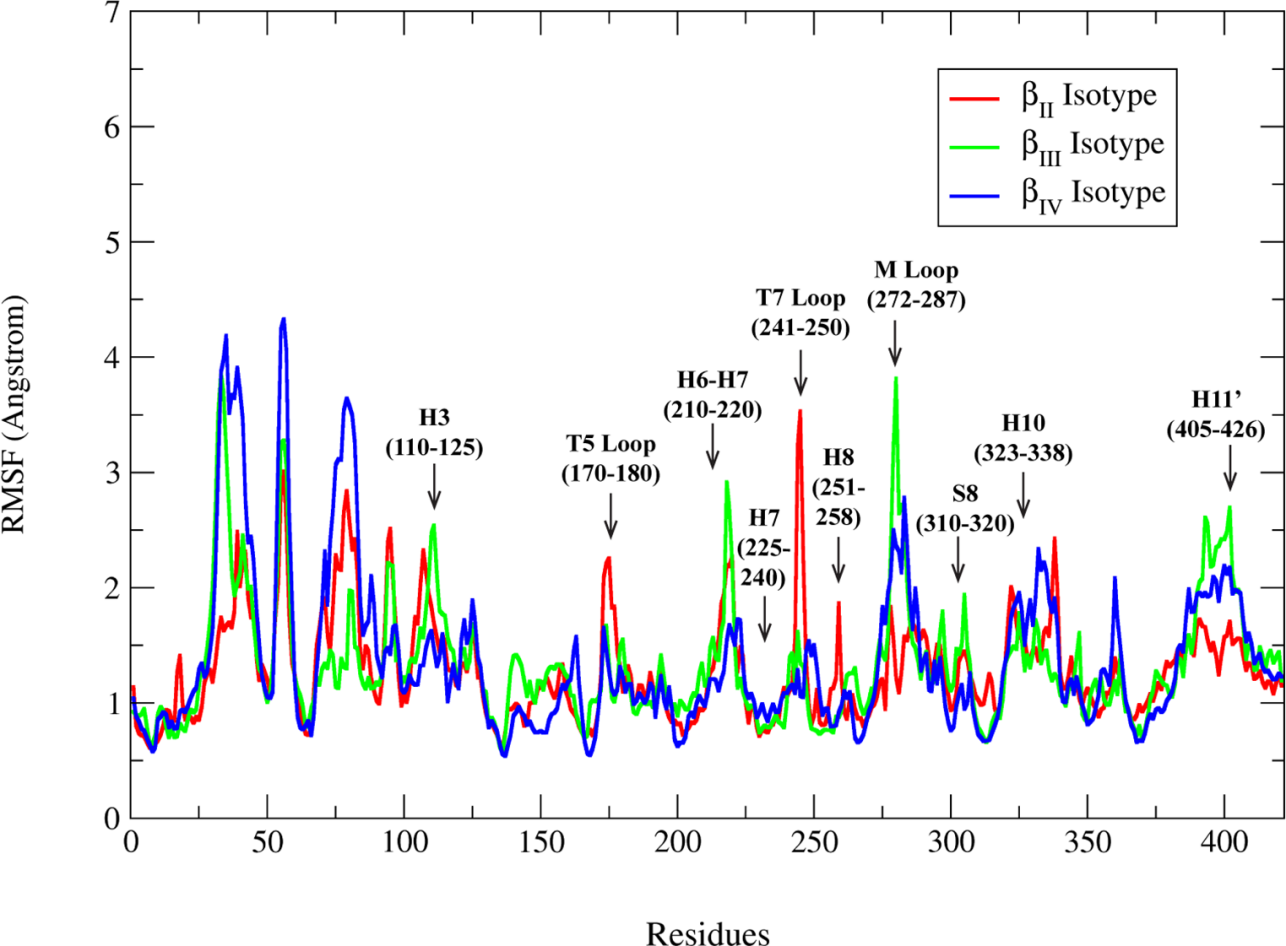
Root mean square fluctuations (RMSF) corresponding to β-tubulin isotypes. Root mean square fluctuations (RMSF) αβ_II_ (red colour), αβ_III_ (green colour) and αβ_IV_ (blue colour) tubulin heterodimer for 25ns MD simulations.

RMSF plots provide information on the flexible regions of the MD simulated structures. RMSF mainly calculates the degree of movement of Cα atoms around their average positions. The highly flexible regions show higher RMSF value while the constrained regions show low RMSF value. We have calculated the RMSF of human tubulin isotypes β_II_, β_III_ and β_IV_ using the PTRAJ module of AMBER 12. All the β-tubulin isotypes show flexibility below the range of 5Å (Fig 5). A comparison of RMSF plots of different isotypes shows that H6-H7 (210–220) helix and M loop (272–287) of β_III_ isotype shows higher fluctuations than β_II_ and β_IV_ isotypes. Hence, for detailed understanding of the bonding and nonbonding interactions of residues at the binding site of DAMA-colchicine, we analyzed the MD simulated structures of tubulin 1SA0 and tubulin isotypes.

### Analysis of MD simulation structures of αβ tubulin-DAMA-colchicine complex

**Fig 6:**
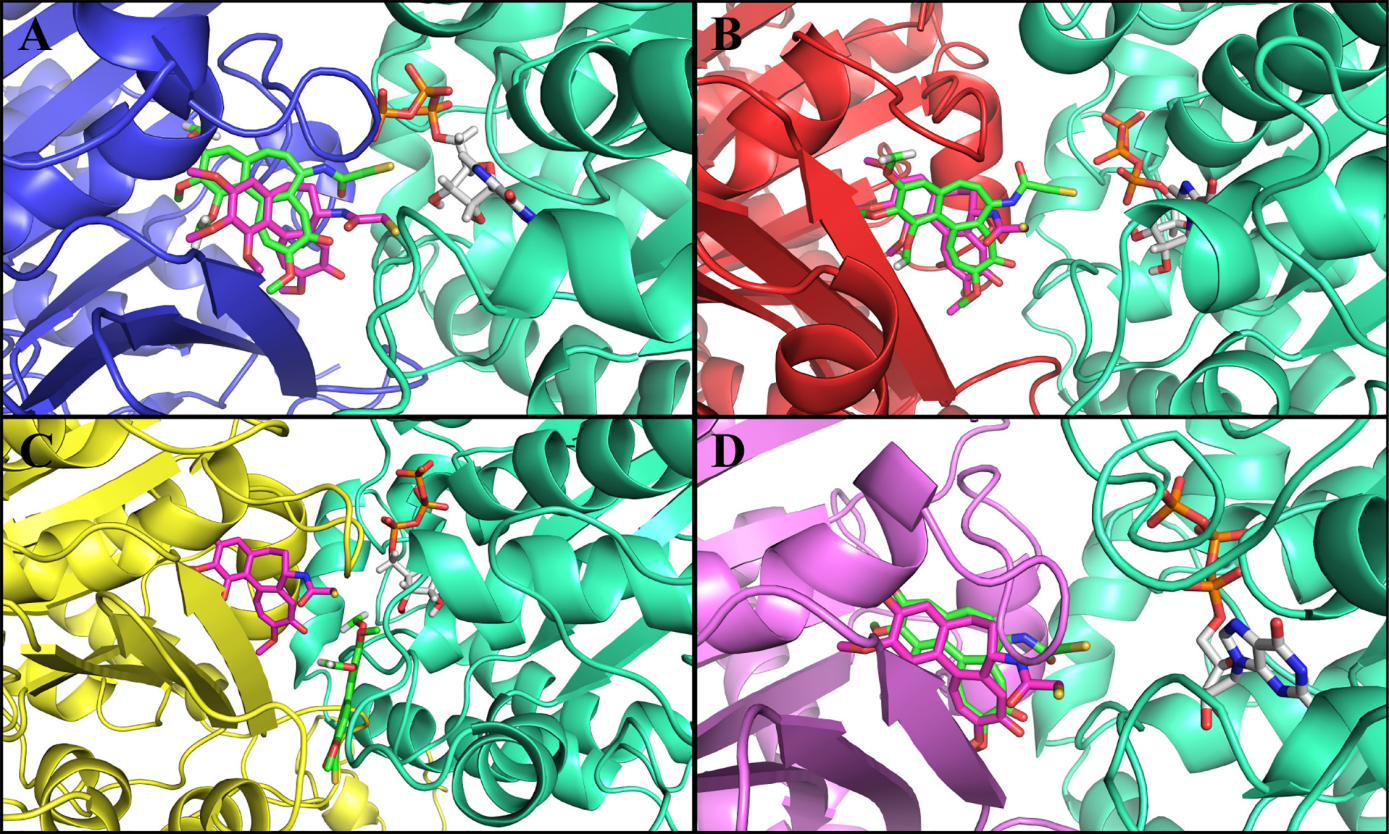
Molecular dynamics (MD) simulated end structures of tubulin 1SA0 and tubulin isotypes. The positions of DAMA-colchicine before and after simulation are shown for comparison. The colour scheme for αβ-tubulin and DAMA-colchicine is same as for the Fig 3. Initial docked conformation of DAMA-colchicine (before simulation) shown in magenta colour while DAMA-colchicine after simulation is shown in green colour similar to Fig 3. (A) Tubulin 1SA0 and DAMA-colchicine complex. (B) αβ_II_ tubulin isotype-DAMA-colchicine complex. (C) αβ_III_ tubulin isotype-DAMA-colchicine complex (D) αβ_IV_ tubulin isotype-DAMA-colchicine complex. It is observed after simulation that the DAMA-colchicine moves away from the initial position in αβ_III_ tubulin isotype.

The hydrogen bonding interactions and residues lying around 4 Å distance of ligand after MD simulations were used to understand the binding mode of DAMA-colchicine. The molecular dynamics simulated structures for each system i.e. tubulin 1SA0, αβ_II_ isotype, αβ_III_ isotype and αβ_IV_ isotype (Fig 6A–6D) were considered for the analysis of bonding and non-bonding residues present around DAMA-colchicine. DAMA-colchicine prefers αβ interface for tubulin 1SA0 (Fig 6A), αβ_II_ (Fig 6B) and αβ_IV_ (Fig 6D) tubulin isotypes, except for the αβ_III_ (Fig 6C) tubulin isotype. In αβ_III_ tubulin, DAMA-colchicine moves from the initial docked position (Fig 3C) and goes to the surface of interface (Fig 6C and S3 Movie). Here, we considered only the RMSD of DAMA-colchicine. The RMSD between molecular dynamics simulated ‘Starting structure’ i.e. docked structure and ‘End structure’ determined binding modes of DAMA-colchicine was 3.59 Å for tubulin 1SA0, 4.32 Å for αβ_II_ isotype, 11.45 Å for αβ_III_ isotype and 2.94 Å for αβ_IV_ isotype (Table 3). Thus, root mean square deviations (RMSD) analysis shows that DAMA-colchicine largely deviates from the starting position in αβ_III_ tubulin isotype (Fig 6C). The hydrogen bonding and residues lying around the 4Å distance of DAMA-colchicine in tubulin 1SA0 and tubulin isotypes are shown in Table 3 and Table 4 respectively.

**Table 3:** RMSD of DAMA-colchicine relative to docked structure, and hydrogen bonding interactions of DAMA-colchicine, with tubulin 1SA0 and tubulin isotypes after MD simulation.

Protein structure	RMSD of DAMA-colchicine after simulation Å	Hydrogen bonding interactions			Figure Reference
		Atoms involved 1-2-3	Bond Distance (Å)	Bond Angle (Degree)	
**Tubulin 1SA0**	3.59	Glu-198 OE2…..HC6-COL	2.35	111.32	6A, Fig A in S8 Fig
		Cys-239 HG…….O1-COL	3.12	126.31	
		Leu-246 HD11….O5-COL	2.37	156.96	
		Leu-253 HD13….O2-COL	3.43	111.44	
		Asn-256 HD21….O4-COL	2.78	118.59	
		Lys-350-HB3……O6-COL	3.20	141.94	
**αβ**_**II**_ **isotype**	4.32	Cys-239-HC…….O3-COL	2.61	140.35	6B, Fig B in S8 Fig
		Leu-246 HD12….N1-COL	3.19	156.52	
		Leu-246-O…….HC6-COL	2.31	155.94	
		Asn-256 HB3……O5-COL	3.35	132.55	
		COL-O3…………Ala-244	2.80	128.89	
**αβ**_**III**_ **isotype**	11.45	Pro-222 HC……. . .O1-COL	2.40	125.75	6C, Fig C in S8 Fig
		Leu-246 HB2…. . .O2-COL	2.71	149.62	
		Asn-348 HD22 …O4-COL	3.10	107.48	
**αβ**_**IV**_ **isotype**	2.94	Leu-246 HD11…..N1-COL	3.26	139.77	6D, Fig D in S8 Fig
		Leu-253 HD13…..O3-COL	2.86	108.23	
		Asn-256 HB3……O-COL	3.40	121.40	
		Lys-350 HG2……O5-COL	3.30	129.75	
		Ala-180-CH……..O4-COL	2.81	143.38	

**Table 4:** Residues present around the 4 Å distances of DAMA-colchicine in tubulin 1SA0 and tubulin isotypes after simulation.

Protein	Residues around 4 Å distance of DAMA-colchicine after simulation
**Tubulin 1SA0**	**β-tubulin residues:** Glu-200, Tyr-202, Gly-235, Val-238, Thr-240, Cys-239, Lys-246, Ala-248, Leu-250, Lys-252, Leu-253, Asn-256, Ala-314, Ala-315, Val-316, Lys-350, Thr-351, Ala-352, Ile-376. **α-tubulin residues:** Asn-101, Ser-178, Thr-179.
**αβ**_**II**_ **isotype**	**β-tubulin residues:** Cys-239, Pro-243, Gly-244, Gln-245, Leu-246, Lys-252, Leu-253, Asn-256, Met-257, Ala-314, Ile-316, Asn-347, Lys-350, Thr-351, Ala-352, Cys-354. **α-tubulin residues:** Thr-179, Ala-180, Val-181.
**αβ**_**III**_ **isotype**	**β-tubulin residues:** Gln-245, Leu-246, Asp-328, Leu-331, Asn-347, Val-349, Lys-350, Val-351, Ala-352. **α-tubulin residues:** Gln-176, Val-177, Ser-178,Thr-179, Ala-180, Val-181, Thr-210,Glu-220, Arg-221.
**αβ**_**IV**_ **isotype**	**β-tubulin residues:** Glu-199, Tyr-200, Val-236, Cys-239, Leu-240, Leu-250, Lys-252, Leu-253, Ala-254, Asn-256, Met-257, Phe-266, Thr-312, Ala-314, Asn-348, Lys-350, Ile-376. **α-tubulin residues:** Asn-101, Ser-178, Thr-179, Ala-180, Val-181.

The analysis of MD simulated representative structure of tubulin 1SA0-DAMA-colchicine complex shows the hydrogen bonding interactions of DAMA-colchicine with Glu-198(2.35Å), Cys-239 (3.12Å), Leu-246 (2.37Å), Leu-253 (3.43Å), Asn-256 (2.78Å) and Lys-350 (3.20Å) as shown in Table 3. Asn-256 and Lys-350 interact with the mercaptoacetyl group; Glu-198, Leu-253 and Cys-239 interact with the trimethoxy benzene ring and Leu-246 with methoxytropone ring of DAMA-colchicine. In tubulin 1SA0, DAMA-colchicine interacts mostly with the β-tubulin residues at the binding pocket (Table 4). The interactions are shown in Fig A in S8 Fig. In the MD simulated αβ_II_ tubulin-DAMA-colchicine complex, DAMA-colchicine shows hydrogen bond with residues Cys-239 (2.61Å), Leu-246 (3.19Å), Leu-246 (2.31Å), Asn-256 (3.35Å) and Ala-244 (2.80Å) of β-tubulin (Table 3 and Fig B in S8 Fig). The Asn-256 interacts with the methoxytropone ring, Leu-246 interact with mercaptoacetyl group and Cys-239 with trimethoxy ring of DAMA-colchicine.

Analysis of αβ_III_ tubulin-DAMA-colchicine complex shows hydrogen bonding of DAMA-colchicine with residues Leu-246(2.71 Å) of β tubulin and Asn-348 (3.10Å) and Pro-222 (2.40Å) of α tubulin in binding pocket (Table 4 and Fig C in S8 Fig). Comparing the residues lying around the 4Å distance of DAMA-colchicine in the initial structure (Table 2) and the MD simulated end structure (Table 4), it is clearly seen that DAMA-colchicine shifts from the initial binding position and moves out to the surface of interface. In this case, the T5 loop of α-tubulin and B9 sheet β-tubulin shows large conformational changes, whereas the T7 loop comes forward resulting into the closing of the binding pocket (S3 Movie).

Finally, analysis of αβ_IV_ tubulin-DAMA-colchicine complex shows hydrogen bonding between DAMA-colchicine and Leu-246 (3.26Å), Leu-253 (2.86Å), Asn-256 (3.40Å) and Lys-350 (3.30Å) of β chain and Ala-180(2.81Å) of α chain (Table 3 and Fig D in S8 Fig). The residues Asn-256 and Lys-350 interact with methoxytropone ring, Leu-248 with mercaptoacetyl group and Leu-253 with the trimethoxy benzene ring. In the tubulin 1SA0 and αβ_IV_ tubulin, common amino acids interacting with DAMA-colchicine are Leu-246, Leu-253, Asn-256 and Lys-350 (Table 3).

Our structural analysis of MD simulated tubulin DAMA-colchicine complexes confirms that DAMA-colchicine mostly interacts with the β-tubulin residues. Overall the residues at the binding pocket such as Cys-239, Leu-246, Asn-256, and Leu-253 and Lys-350 of β-tubulin could play an important role in the stabilization of DAMA-colchicine binding. While in αβ_III,_ DAMA-colchicine doesn’t show any such interactions with these residues (Table 3). Hence, we considered only β-tubulin and DAMA-colchicine for electrostatic contact potential calculation to understand the binding mode of DAMA-colchicine after molecular dynamics simulation. The electrostatic potential surfaces show that DAMA-colchicine is located inside the binding cavity of β-tubulin in case of tubulin 1SA0 (Fig A in S9 Fig), αβ_II_ tubulin isotype (Fig B in S9 Fig) and αβ_IV_ tubulin isotype (Fig D in S9 Fig), whereas in αβ_III_ tubulin DAMA-colchicine moves from the initial binding pocket (Fig C in S9 Fig).

The electrostatic and van der Waals interactions along with the hydrogen bonding interactions could also play an important role in the stabilization of the protein-ligand complex. Hence, we further employed MM-GBSA binding free energy calculations, to understand binding free energy differences for tubulin 1SA0 and different tubulin isotypes for DAMA-colchicine.

### Binding Free Energy Calculation

The binding free energy for the tubulin 1SA0 and different tubulin isotypes are shown in Table 5. As stated earlier, the entropic contribution to the binding free energy was ignored in these calculations.

**Table 5:** Binding free energy of tubulin 1SA0, αβ_II_, αβ_III_, and αβ_IV_ tubulin isotypes with DAMA-colchicine.

Protein	Δ*E*_*vdw*_	Δ*E*_*ele*_	Δ*E*_*gas*_	Δ*E*_*sol*_	^*a*^Δ*E*_*bind*_
**Tubulin 1SA0**	-62.99	-1327.85	-1390.84	1334.73	-56.11
**αβ**_**II**_ **isotype**	-54.06	-2045.11	-2099.17	2042.43	-56.74
**αβ**_**III**_ **isotype**	-54.27	-1414.59	-1468.86	1416.91	-51.95
**αβ**_**IV**_ **isotype**	-64.28	-1549.38	-1613.66	1549.21	-64.45

^*a*^Δ*E*_*bind*_ = Δ*E*_*gas*_ + Δ*E*_*sol*_ = (Δ*E*_*vdw*_ + Δ*E*_*ele*_) + (Δ*E*_*polar*_ + Δ*E*_*nonpolar*_)

The estimated binding free energies (Δ*E*_*bind*_) of complexes of DAMA-colchicine with αβ_II_, αβ_III_ and αβ_IV_ tubulin isotype heterodimers are, -56.74, -51.95 and -64.45 kcal/mol, respectively (Table 5). The αβ_IV_ tubulin isotype has the highest binding free energy for DAMA-colchicine, whereas αβ_III_ tubulin isotype has the lowest binding free energy among three isotypes (Table 5). The binding free energy (Δ*E*_*bind*_) of tubulin isotype for DAMA-colchicine decreases in the order αβ_IV_ > αβ_II_ > αβ_III_. The order of decrease is similar to experimentally measured binding energies for colchicine as well as Desacetamidocolchicine for these three isotype from bovine (Text B and Text C in S1 Text) [2, 25]. Relative differences in binding energies are similar to ones measured for Desacetamidocolchicine yet they are very different from ones measured for colchicine (αβ_IV_ > αβ_II ≃_ αβ_III_). Thus, changes in residue composition at the DAMA-colchicine binding site of αβ_II_ and αβ_III_ tubulin isotype have an impact on the binding free energy (Δ*E*_*bind*_).

The electrostatic (Δ*E*_*ele*_) and van der Waal (Δ*E*_*vdw*_) interactions play a prominent role in binding free energy of protein-ligand complex. The electrostatic energy (Δ*E*_*ele*_) is favorable for binding; the αβ_III_ tubulin isotype shows lowest electrostatic interaction energy (Δ*E*_*ele*_) in comparison to αβ_II_ and αβ_IV_ isotypes. Here, the electrostatic free energy makes the greatest contribution to the binding free-energy (Table 5). The solvation energy (*E*_*sol*_) consists of polar and non-polar contributions and is unfavorable for binding. The net binding free energy, which is decided by the competition of *E*_*gas*_ and *E*_*sol*_, is lowest for αβ_III_ tubulin isotype. Thus, the binding free energy calculation supports the results obtained from molecular docking and MD simulation studies that the tubulin isotype αβ_III_ shows less binding affinity for DAMA-colchicine. Binding free energies reported here should not be compared directly with the ones obtained experimentally, as our free energy calculations do not include entropic contributions.

### Understanding the Effect of residue variations present in αβ_III_ Tubulin Isotype

It is clear from the docking, molecular dynamics simulation and binding energy calculations that DAMA-colchicine is repelled from the interface of αβ_III_ tubulin isotype (Figs 3 and 6 and Table 5). The colchicine binding pocket of αβ_III_ tubulin isotype has three residue changes Ser-239, Thr315, and Val351. However, it is not clear as to which residues among Ser-239, Thr-315 and Val-351 at the colchicine binding pocket play a major role in affecting the binding of colchicine to αβ_III_ tubulin isotype. In the tubulin 1SA0, amino acid Cys-239 of β-tubulin is involved in β-tubulin recognition site for colchicine [48]. Sulfhydryl group of Cys-239 present in β_I_, β_II_ and β_IV_ isotypes of bovine tubulin is readily oxidized and its oxidation inhibits microtubule assembly [49]. Hence, change of Cys-239 to Ser in αβ_III_ isotype could play a role in affecting the binding of colchicine. In αβ_III_ isotype, there are two other residue changes, Thr315 and Val351 in B8 and B9 beta sheet respectively, which may cause disruption in interactions with helices 9 and 10. Thus, these residue changes in the beta sheet near the colchicine binding region may also affect the colchicine binding. To get a better understanding as to which residues play a major role in the binding of DAMA-colchicine, each of these three residues Ser-239, Thr-315 and Val-351 were in-silico mutated back to the residues found in the tubulin 1SA0 i.e. Cys-239, Ala-315 and Thr-351.

Here, we built four different systems with in-silico mutations: (1) αβ_III_ isotype with Ser239 to Cys (2) αβ_III_ isotype with Thr315 to Ala (3) αβ_III_ isotype Val351 to Thr and (4) αβ_III_ isotype with Ser239-Cys, Thr315-Val and Thr-351-Val similar to tubulin 1SA0 (details of molecular modeling in Text D in S1 Text). The RMSD between the ‘predicted’ and ‘crystallographically’ determined binding modes of DAMA-colchicine, using molecular docking studies, for these four in-silico mutant structures are listed in Table 6. The RMSD of the DAMA-colchicine in these in-silico mutant structures of αβ_III_ isotypes such as Ser239-Cys (Fig A in S10 Fig), Thr315-Ala (Fig B in S10 Fig), Val351-Thr (Fig C in S10 Fig) and Ser239-Cys, Thr315-Ala Val351-Thr (Fig D in S10 Fig) were 9.50 Å, 7.56 Å, 7.69 Å and 2.08 Å, respectively. The RMSD in the range of 2–3 Å indicates appropriate binding [27, 36]. Hence, the RMSD value shows that all residues at the colchicine binding pocket (Ser-239, Thr315, and Val351) play a role in affecting the binding of DAMA-colchicine to αβ_III_ isotype. Further, we also compared the binding energy difference of the minimum energy docked conformations of DAMA-colchicine (Table 6) to these in-silico mutated structures. The binding energy of minimum energy docked DAMA-colchicine with Ser239-Cys (Fig A in S10 Fig), Thr315-Ala (Fig B in S10 Fig), Val351-Thr (Fig C in S10 Fig) and Ser239-Cys, Thr315-Ala Val351-Thr (Fig D in S10 Fig) in-silico mutant structures are -7.95, -6.92, -6.81 and -9.00 kcal/mol, respectively (Table 6). Hence, the RMSD and binding energy results show that all the mutations Ser-239, Thr-315 and Val-351 collectively affect the binding of DAMA-colchicine to αβ_III_ isotype.

**Table 6:** RMSD of DAMA-colchicine relative to crystal structure, binding energy and hydrogen bonding interactions of DAMA-colchicine with in-silico mutant structures of αβ_III_ tubulin isotypes after docking.

In-silico Mutant structure	RMSD of DAMA-colchicine	^a^Binding energy (kcal mol^-1^)	Figure reference
**Ser-239-Cys**	9.50	-7.95	Fig A in S10 Fig
**Thr315-Ala**	7.56	-6.92	Fig B in S10 Fig
**Val351-Thr**	7.69	-6.81	Fig C in S10 Fig
**Ser239-Cys, Thr315-Ala, Val351-Thr**	2.08	-9.00	Fig D in S10 Fig

^a^Binding energy values are obtained from the lowest energy DAMA-colchicine docked complex.

## Conclusions

In this study, we investigated the differential binding affinity of human tubulin isotypes αβ_II_, αβ_III_ and αβ_IV_ towards DAMA-colchicine using molecular docking, molecular dynamics simulation and binding free energy calculations. The sequence analysis of β-tubulin isotypes showed presence of residue changes at the binding site of isotype β_II_ and β_III_ that may affect the binding of DAMA-colchicine. The docking results show that DAMA-colchicine prefers the αβ interface of tubulin in tubulin 1SA0, αβ_II_ and αβ_IV_ isotype as similar to the crystal structure, whereas it prefers a different orientation in αβ_III_ isotype. After docking, the RMSD values for ‘predicted’ and ‘crystal structure’ of DAMA-colchicine in αβ_II,_ αβ_III_ and αβ_IV_ tubulin isotypes are 2.4Å, 5.2Å and 2.1Å respectively, indicating that residue changes at the binding site of αβ_III_ affect the binding of DAMA-colchicine. We further find that Lys-350 forms hydrogen bond with methoxytropone ring of DAMA-colchicine in αβ_II_ and αβ_IV_, whereas Lys-350 forms hydrogen bond with mercaptoacetyl group of DAMA-colchicine in αβ_III_. The hydrogen bonding of Lys-350 with mercaptoacetyl group alters the binding pose of the DAMA-colchicine in the binding pocket in αβ_III_. This could explain the order of binding affinities (αβ_IV ≃_ αβ_II_ >> αβ_III_) observed for DAMA-colchicine which is different from experimentally measured binding energies of tubulin isotypes for colchicine (αβ_IV_ > αβ_II_ ~ αβ_III_).

Molecular dynamics (MD) simulations were performed on tubulin-DAMA-colchicine docked conformation to further investigate the differential binding affinity of DAMA-colchicine for tubulin isotypes. Our MD simulations show that DAMA-colchicine is adapted inside the β-tubulin binding site in tubulin isotypes αβ_II_ and αβ_IV_, whereas DAMA-colchicine is expelled from the binding pocket in the αβ_III_ tubulin isotype. The RMSD of the initial and final binding poses of DAMA-colchicine after MD simulation in αβ_II,_ αβ_III_ and αβ_IV_ tubulin isotypes are 4.32Å, 11.45Å and 2.94Å respectively.

Further, the electrostatic and van der Waals interactions play important roles in the binding of DAMA-colchicine at αβ tubulin interface in tubulin 1SA0 and αβ_IV_, whereas the loss of these interactions in αβ_II_ and αβ_III_ may affect the binding. In addition, the docking study of αβ_III_ with in-silico mutations confirms that, all three changed residues at the binding site of αβ_III_ isotype are collectively responsible for affecting the binding of DAMA-colchicine.

Our computational studies not only provide a detailed understanding of differential binding affinity of DAMA-colchicine for tubulin isotypes but they also provides another example of how sensitive the binding of colchicine congeners is to alternations of the side chain of the B-ring. This detailed understanding of the tubulin isotype and DAMA-colchicine interactions will provide some useful insights for designing better analogues in future.

## Supporting Information

S1 Fig **(A) PROCHECK plot (B) Verify-3D plot and (C) ERRAT plot for tubulin 1SA0.** The PROCHECK result shows that 76.5% residues are in favored regions, 18% residues in additional allowed regions, 4.1% of residues in generously allowed regions and 1.3% residues in disallowed regions. The red region in Ramachandran plot is ‘most favoured’, bright yellow is ‘additional allowed’, dull yellow is the ‘generously allowed’ and white is the ‘disallowed’ region. The VERIFY-3D score was 97.86, and the ERRAT score was 77.25, further indicating the good quality of the template model (Tubulin 1SA0.pdb).
(PDF)

S2 Fig **(A) PROCHECK plot (B) Verify-3D plot and (C) ERRAT plot for αβ**_**II**_ **tubulin isotype.** The PROCHECK result shows that 87.1% residues are in favored regions, 10.5% residues in additional allowed regions, 1.3% of residues in generously allowed regions and 1.0% residues in disallowed regions. The red region in Ramachandran plot is ‘most favoured’, bright yellow is ‘additional allowed’, dull yellow is the ‘generously allowed’ and white is the ‘disallowed’ region. The VERIFY-3D score was 95.81% and the ERRAT score was 88.05, further indicating the good quality of the model of human αβ_II_ tubulin isotype.
(PDF)

S3 Fig **(A) PROCHECK plot (B) Verify-3D plot and (C) ERRAT plot for αβ**_**III**_ **tubulin isotype.** The PROCHECK result shows that 87.5% residues are in favored regions, 10.1% residues in additional allowed regions, 1.4% of residues in generously allowed regions and 1.0% residues in disallowed regions. The red region in Ramachandran plot is ‘most favoured’, bright yellow is ‘additional allowed’, dull yellow is the ‘generously allowed’ and white is the ‘disallowed’ region. The VERIFY-3D score was 95.25% and the ERRAT score was 88.60, further indicating the good quality of the model of human αβ_III_ tubulin isotype.
(PDF)

S4 Fig **(A) PROCHECK plot (B) Verify-3D plot and (C) ERRAT plot for αβ**_**IV**_ **tubulin isotype.** The PROCHECK result shows that 86.6% residues are in favored regions, 11.4% residues in additional allowed regions, 1.2% of residues in generously allowed regions and 0.8% residues in disallowed regions. The red region in Ramachandran plot is ‘most favoured’, bright yellow is ‘additional allowed’, dull yellow is the ‘generously allowed’ and white is the ‘disallowed’ region. The VERIFY-3D score was 94.99% and the ERRAT score was 89.85, further indicating the good quality of the model of human αβ_IV_ tubulin isotype.
(PDF)

S5 Fig Hydrogen bonding interactions of DAMA-colchicine with tubulin 1SA0 and tubulin isotypes after docking. Color scheme for α-tubulin is green_cyan and β-tubulin is tv_blue for tubulin 1SA0, tv_red for isotype β_II_, tv_yellow for isotype β_III_ and violet for isotype β_IV_. Crystal structure of DAMA-colchicine after docking is shown in green color. The oxygen, nitrogen and sulphur atoms of DAMA-colchicine has are shown in red, blue and pale yellow color, respectively. The hydrogen bonds are shown as black dotted line between tubulin residues and DAMA-colchicine, (A) Hydrogen bonding between DAMA-colchicine (green color) and tubulin 1SA0 residues i.e. Cys-239(2.20Å), Lys-350(2.31Å) and Val-181(1.91Å) at the interface binding pocket. (B) Hydrogen bonding between DAMA-colchicine and αβ_II_ tubulin isotype residues i.e. Lys-350(2.07Å), Cys-239(2.05Å) and Asn-256(2.15Å) at binding pocket. (C) Hydrogen bonding interaction between with DAMA-colchicine and αβ_III_ tubulin isotype residues i.e. Lys-350(2.65Å) of β-tubulin and Asn-101(2.20Å) α-tubulin. (D) Hydrogen bonding between DAMA-colchicine and αβ_IV_ tubulin isotype i.e. Cys-239(2.08Å) and Lys-352(2.25Å) in the binding pocket.
(TIF)

S6 Fig The electrostatic contact potential of tubulin 1SA0 and β-tubulin isotypes with docked DAMA-colchicine. The red, blue and white color represents the negative, positive and neutral electrostatic potentials, respectively. The drug DAMA-colchicine bind at the interface of cavity of β-tubulin in tubulin 1SA0 and tubulin isotypes. DAMA-colchicine is shown in green color;oxygen, nitrogen, and sulphur atoms are shown in red, blue, and golden yellow colors respectively. **(A)** β-tubulin 1SA0 and DAMA-colchicine complex **(B)** β_II_ tubulin isotype and DAMA-colchicine complex, **(C)** β_III_ tubulin isotype and DAMA-colchicine complex **(D)** β_IV_ tubulin isotype and DAMA-colchicine complex. In β_III_ tubulin isotype, DAMA-colchicine prefers different conformation.
(TIF)

S7 Fig Radius of gyration for tubulin 1SA0 and β-tubulin isotypes. Radius of gyration (Rg) values correspond to (A) tubulin 1SA0 (black colour), (B) β_II_ (red colour), (C) β_III_ (green colour) and (D) β_IV_ (blue colour) of tubulin for 25ns MD simulations. The radius of gyration shows the compactness and stable behavior of β-tubulin structures, during molecular dynamics simulations, for tubulin 1SA0 and three β-tubulin isotypes.
(TIF)

S8 Fig Hydrogen bonding after molecular dynamics simulation in tubulin 1SA0 and tubulin isotype. The residues involved in bonding have been shown in stick with cyan color. Hydrogen bonding between DAMA-colchicine and tubulin residues is shown with black dotted line. Crystal structure of DAMA-colchicine after molecular dynamics simulation is shown in green color. The oxygen, nitrogen and sulphur atoms of DAMA-colchicine has are shown in red, blue and pale yellow color, respectively. **(A)** Hydrogen bonding between DAMA-colchicine and tubulin 1SA0 amino acids i.e. Glu-198(2.35Å), Cys-239 (3.12Å), Leu-253 (3.43Å), Asn-256 (2.78Å) and Lys-350 (3.20Å) at binding pocket. **(B)** Hydrogen bonding between DAMA-colchicine and αβ_II_ tubulin isotype amino acids i.e. Cys-239(2.61Å), Leu-246 (3.19Å), Leu-246 (2.31Å) Asn-258 (3.35Å) and Ala-244 (2.80Å). **(C)** Hydrogen bonding between DAMA-colchicine and αβ_III_ isotype amino acids i.e. Leu-246(2.71 Å) of β tubulin and Asn-348 (2.78Å) and Pro-222 (2.40Å) of α tubulin in binding pocket. **(D)** Hydrogen bonding interactions between DAMA-colchicine and αβ_IV_ isotype residues i.e. Leu-246 (3.26Å), Leu-253 (2.86Å), Asn-256 (3.40Å) and Lys-350 (3.30Å) of β chain and Ala-180(2.81Å) of α chain.
(TIF)

S9 Fig The electrostatic contact potential of DAMA-colchicine with tubulin 1SA0 and β-tubulin isotypes after molecular dynamics simulation. Colour scheme is same as shown in S6 Fig. (A) β-tubulin 1SA0 and DAMA-colchicine (B) β_II_ tubulin isotype and DAMA-colchicine C) β_III_ tubulin isotype and colchincine and (D) β_IV_ tubulin isotype and DAMA-colchicine. After MD simulation, the drug DAMA-colchicine located inside the binding pocket of β-tubulin in tubulin 1SA0 (A), β_II_ (B) and β_IV_ (D) isotypes whereas in β_III_ isotypes (C), it expelled from the binding pocket.
(TIF)

S10 Fig The conformation of DAMA-colchicine after docking with in-silico mutant structures of αβ_III_ tubulin isotypes. Color scheme for α-tubulin is tv_orange and β-tubulin is aquamarine. The conformation of DAMA-colchicine (shown in magenta) after docking with in-silico mutant structures of αβ_III_ tubulin isotype (A) αβ_III_ isotype with Ser 239 to Cys mutation, (B) αβ_III_ isotype with Thr 315 to Ala, (C) αβ_III_ isotype with Val 351 to Thr, (D) αβ_III_ isotype with Ser 239-Cys, Thr315-Ala and Val351-Thr. The binding pose of DAMA-colchicine in crystal structure is shown in green.
(TIF)

S1 Movie MD simulation movie of tubulin 1SA0 and DAMA-colchicine. (MPG)

S2 Movie MD simulation movie of αβ_II_ tubulin isotype and DAMA-colchicine. (MPG)

S3 Movie MD simulation movie of αβ_III_ tubulin isotype and DAMA-colchicine. (MPG)

S4 Movie MD simulation movie of αβ_IV_ tubulin isotype and DAMA-colchicine. (MPG)

S1 Text (A) Quality of Homology models of tubulin isotypes. (B) Experimentally measured binding energies of colchicine for bovine αβ_II_ αβ_III_ and αβ_IV_ tubulin isotypes. (C) Experimentally measured binding energies of Desacetamidocolchicine (DAAC), a fast binding analogue of colchicine for bovine αβ_II_ αβ_III_ and αβ_IV_ tubulin isotypes. (D) Molecular modeling and docking study of in-silico mutant structures of αβ_III_ tubulin isotypes and DAMA-colchicine.
(PDF)
