# Supplementary material for: Exploring the Origin of Differential Binding Affinities of Human Tubulin Isotypes αβII, αβIII and αβIV for DAMA-Colchicine Using Homology Modelling, Molecular Docking and Molecular Dynamics Simulations
Source: PLoS One. 2016 May 26;11(5):e0156048. doi: 10.1371/journal.pone.0156048 (PMC4882049; doi:10.1371/journal.pone.0156048)
Supplement: S1 Fig — (A) PROCHECK plot (B) Verify-3D plot and (C) ERRAT plot for tubulin 1SA0. The PROCHECK result shows that 76.5% residues are in favored regions, 18% residues in additional allowed regions, 4.1% of residues in generously allowed regions and 1.3% residues in disallowed regions. The red region in Ramachandran plot is ‘most favoured’, bright yellow is ‘additional allowed’, dull yellow is the ‘generously allowed’ and white is the ‘disallowed’ region. The VERIFY-3D score was 97.86, and the ERRAT score was 77.25, further indicating the good quality of the template model (Tubulin 1SA0.pdb). (PDF) [file pone.0156048.s001.pdf]

S1 Fig.

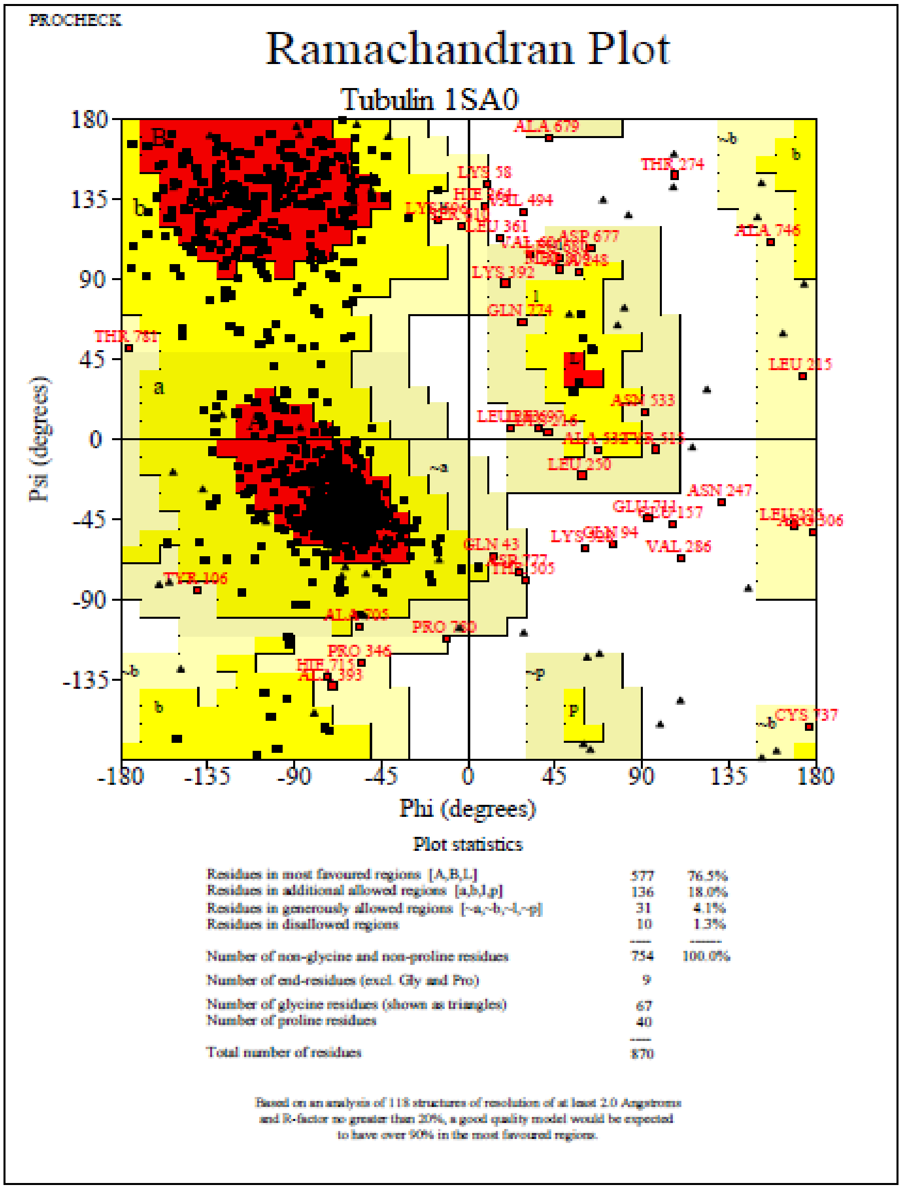
PROCHECK
Ramachandran Plot
Tubulin 1SA0
Psi (degrees)
Phi (degrees)
180
135
90
45
0
-45
-90
-135
-180
Plot statistics
Residues in most favoured regions [A,B,L] 577 76.5%
Residues in additional allowed regions [a,b,l,p] 136 18.0%
Residues in generously allowed regions [~a,~b,~l,~p] 31 4.1%
Residues in disallowed regions 10 1.3%
Number of non-glycine and non-proline residues 754 100.0%
Number of end-residues (excl. Gly and Pro) 9
Number of glycine residues (shown as triangles) 67
Number of proline residues 40
Total number of residues 870
Based on an analysis of 118 structures of resolution of at least 2.0 Angstroms
and R-factor no greater than 20%, a good quality model would be expected
to have over 90% in the most favoured regions.

(A)

Verify 3D graph of Tubulin 1SA0

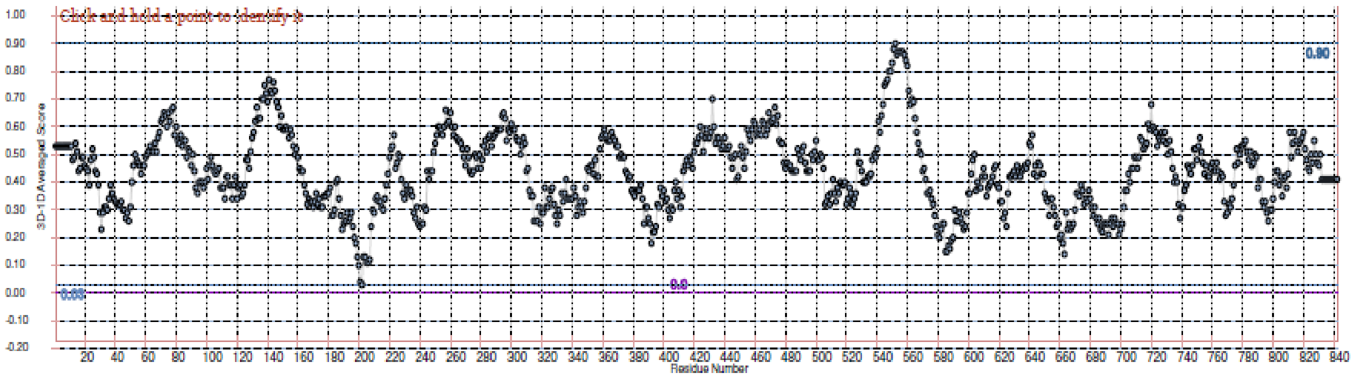

(B)

Program: ERRAT2
File: /var/www/SAVES/Jobs/5337210//errat.pdb
Chain#:1
Overall quality factor**: 77.251

Errat score of Tubulin 1SA0

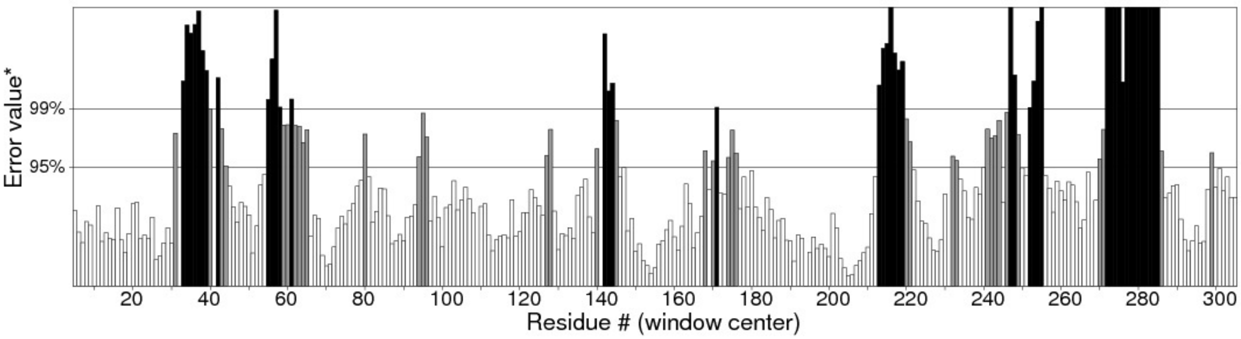

*On the error axis, two lines are drawn to indicate the confidence with which it is possible to reject regions that exceed that error value.

**Expressed as the percentage of the protein for which the calculated error value falls below the 95% rejection limit. Good high resolution structures generally produce values around 95% or higher. For lower resolutions (2.5 to 3A) the average overall quality factor is around 91%.

(C)
