# Supplementary material for: Exploring the Origin of Differential Binding Affinities of Human Tubulin Isotypes αβII, αβIII and αβIV for DAMA-Colchicine Using Homology Modelling, Molecular Docking and Molecular Dynamics Simulations
Source: PLoS One. 2016 May 26;11(5):e0156048. doi: 10.1371/journal.pone.0156048 (PMC4882049; doi:10.1371/journal.pone.0156048)
Supplement: S3 Fig — (A) PROCHECK plot (B) Verify-3D plot and (C) ERRAT plot for αβIII tubulin isotype. The PROCHECK result shows that 87.5% residues are in favored regions, 10.1% residues in additional allowed regions, 1.4% of residues in generously allowed regions and 1.0% residues in disallowed regions. The red region in Ramachandran plot is ‘most favoured’, bright yellow is ‘additional allowed’, dull yellow is the ‘generously allowed’ and white is the ‘disallowed’ region. The VERIFY-3D score was 95.25% and the ERRAT score was 88.60, further indicating the good quality of the model of human αβIII tubulin isotype. (PDF) [file pone.0156048.s003.pdf]

S3 Fig.

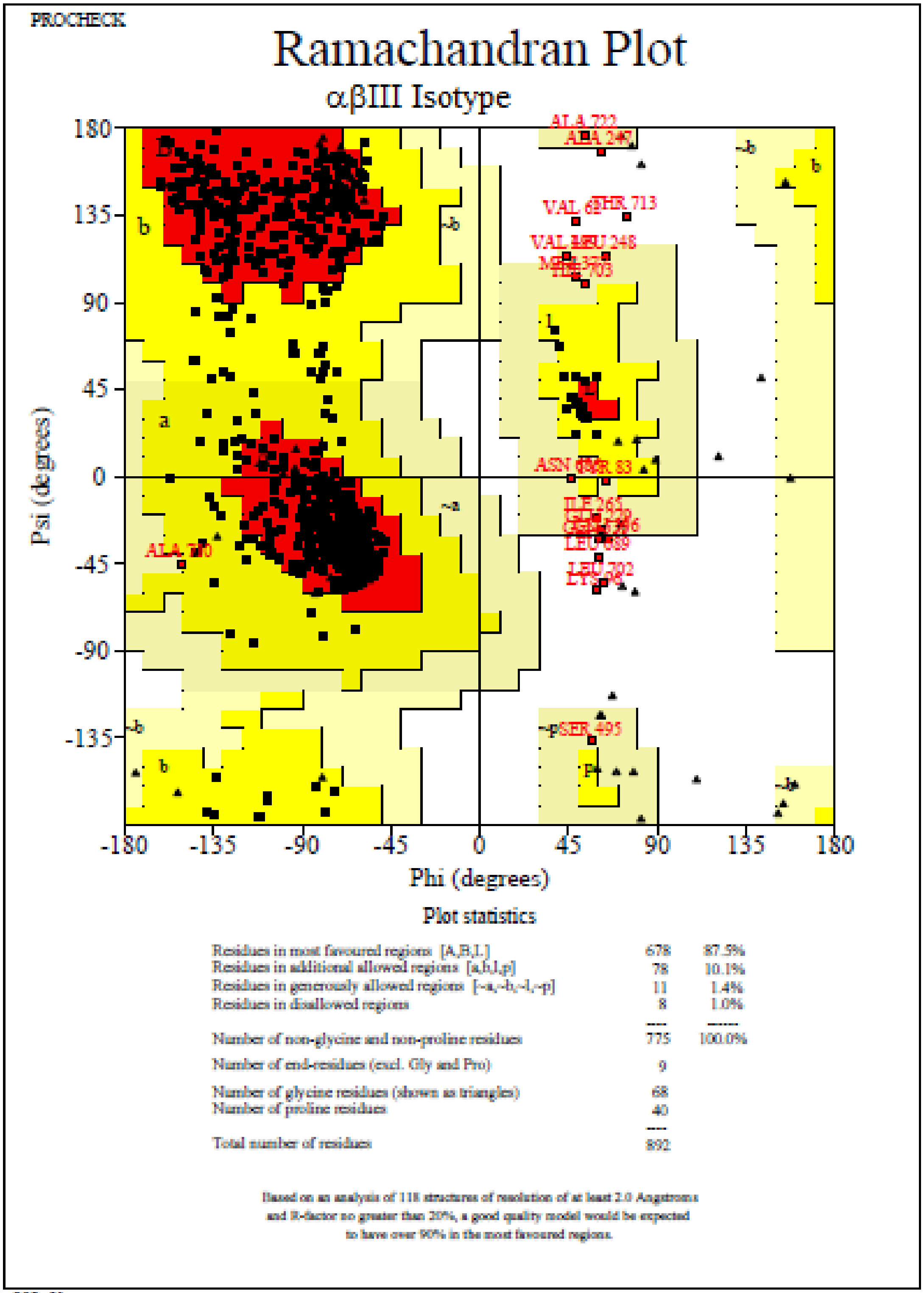

a908_01.ps

(A)

Verify-3D graph of αβIII Isotype

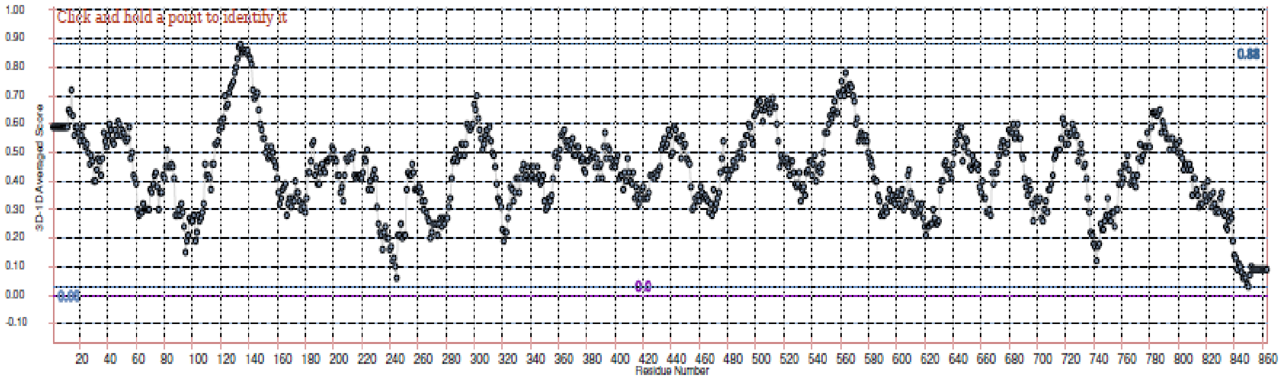

(B)

Program: ERRAT2
File: /var/www/SAVES/Jobs/8685051//errat.pdb
Chain#:1
Overall quality factor**: 88.604

Errat score for αβIII Isotype

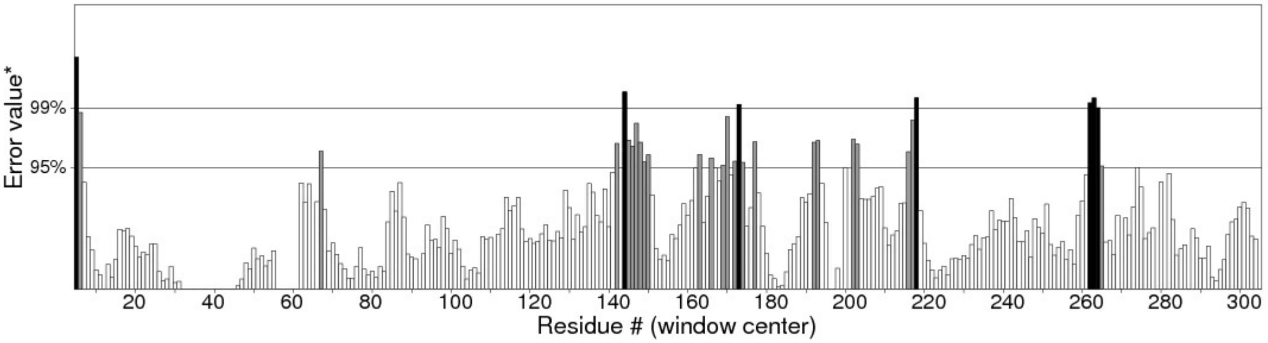

*On the error axis, two lines are drawn to indicate the confidence with which it is possible to reject regions that exceed that error value.

**Expressed as the percentage of the protein for which the calculated error value falls below the 95% rejection limit. Good high resolution structures generally produce values around 95% or higher. For lower resolutions (2.5 to 3A) the average overall quality factor is around 91%.

(C)
