# Supplementary material for: Exploring the Origin of Differential Binding Affinities of Human Tubulin Isotypes αβII, αβIII and αβIV for DAMA-Colchicine Using Homology Modelling, Molecular Docking and Molecular Dynamics Simulations
Source: PLoS One. 2016 May 26;11(5):e0156048. doi: 10.1371/journal.pone.0156048 (PMC4882049; doi:10.1371/journal.pone.0156048)
Supplement: S4 Fig — (A) PROCHECK plot (B) Verify-3D plot and (C) ERRAT plot for αβIV tubulin isotype. The PROCHECK result shows that 86.6% residues are in favored regions, 11.4% residues in additional allowed regions, 1.2% of residues in generously allowed regions and 0.8% residues in disallowed regions. The red region in Ramachandran plot is ‘most favoured’, bright yellow is ‘additional allowed’, dull yellow is the ‘generously allowed’ and white is the ‘disallowed’ region. The VERIFY-3D score was 94.99% and the ERRAT score was 89.85, further indicating the good quality of the model of human αβIV tubulin isotype. (PDF) [file pone.0156048.s004.pdf]

S4 Fig.

PROCHECK

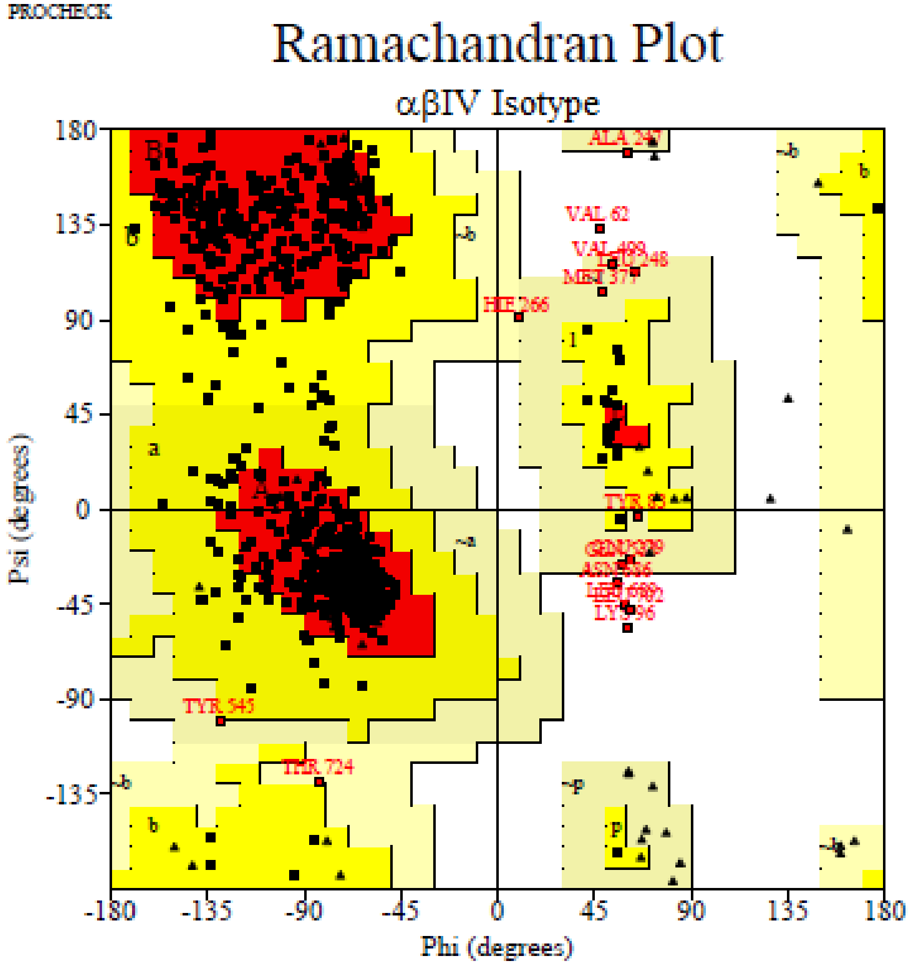

Plot statistics

Residues in most favoured regions [A,B,L]	666	86.6%
Residues in additional allowed regions [a,b,l,p]	88	11.4%
Residues in generously allowed regions [~a,~b,~l,~p]	9	1.2%
Residues in disallowed regions	6	0.8%
	----	------
Number of non-glycine and non-proline residues	769	100.0%
Number of end-residues (excl. Gly and Pro)	9	
Number of glycine residues (shown as triangles)	69	
Number of proline residues	40	

Total number of residues	887	

Based on an analysis of 118 structures of resolution of at least 2.0 Angstroms and R-factor no greater than 20%, a good quality model would be expected to have over 90% in the most favoured regions.

a907_01.ps

(A)

Verify-3D graph of αβIV Isotype

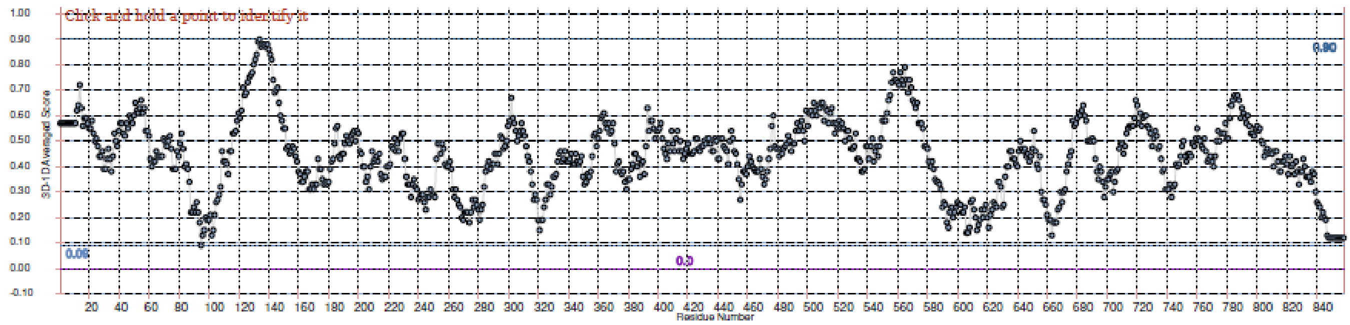

(B)

Program: ERRAT2
File: /var/www/SAVES/Jobs/7149809//errat.pdb
Chain#:1
Overall quality factor**: 89.846

Errat Score of αβIV Isotype

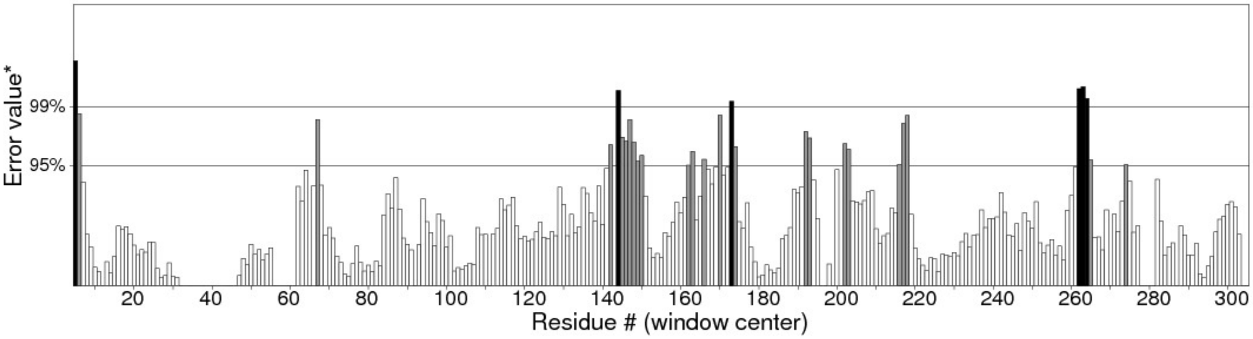

*On the error axis, two lines are drawn to indicate the confidence with which it is possible to reject regions that exceed that error value.

**Expressed as the percentage of the protein for which the calculated error value falls below the 95% rejection limit. Good high resolution structures generally produce values around 95% or higher. For lower resolutions (2.5 to 3A) the average overall quality factor is around 91%.

(C)
