# Supplementary figures and images for: Exploring the Origin of Differential Binding Affinities of Human Tubulin Isotypes αβII, αβIII and αβIV for DAMA-Colchicine Using Homology Modelling, Molecular Docking and Molecular Dynamics Simulations
Source: PLoS One. 2016 May 26;11(5):e0156048. doi: 10.1371/journal.pone.0156048 (PMC4882049; doi:10.1371/journal.pone.0156048)

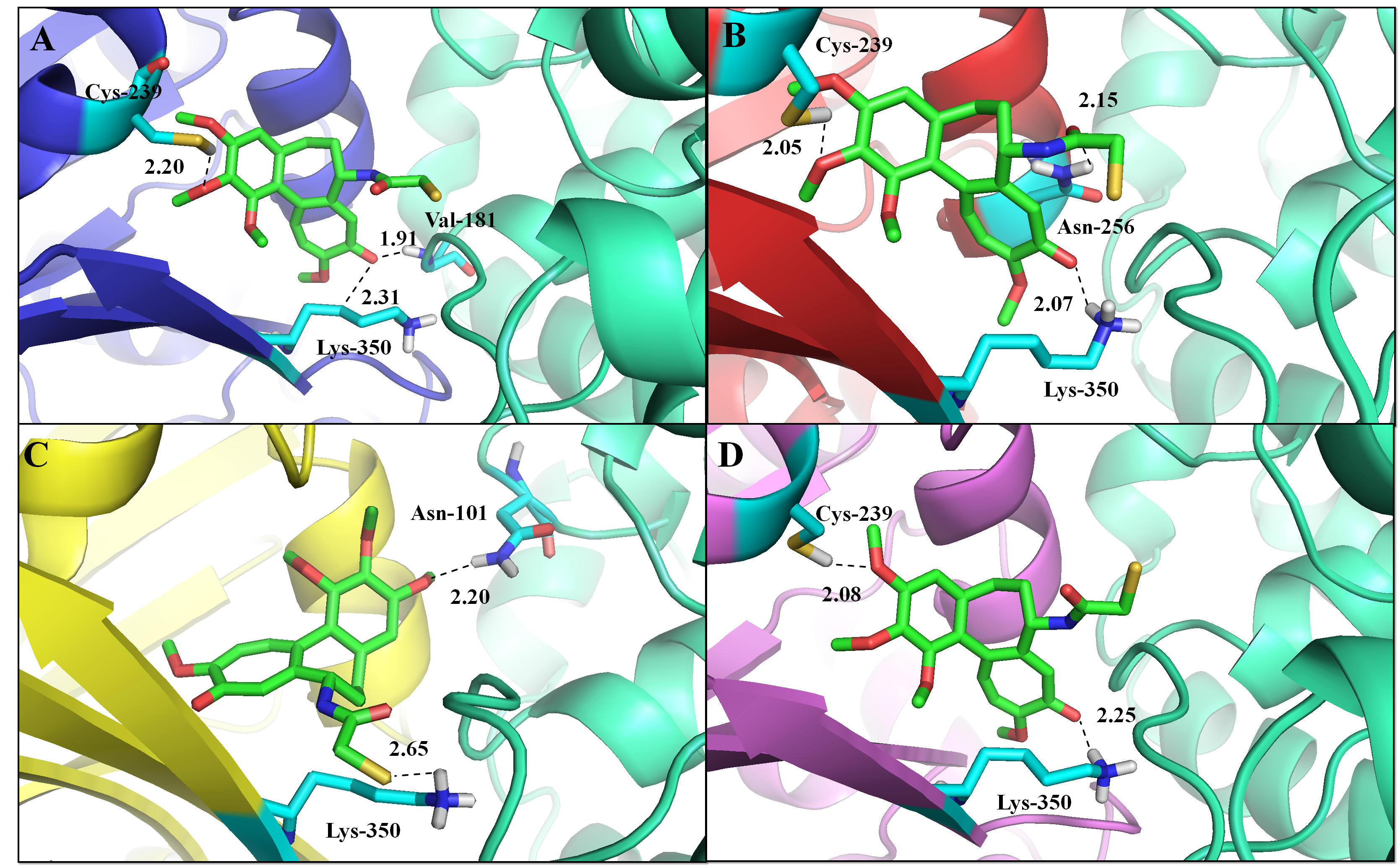

Supplement: S5 Fig — Color scheme for α-tubulin is green_cyan and β-tubulin is tv_blue for tubulin 1SA0, tv_red for isotype βII, tv_yellow for isotype βIII and violet for isotype βIV. Crystal structure of DAMA-colchicine after docking is shown in green color. The oxygen, nitrogen and sulphur atoms of DAMA-colchicine has are shown in red, blue and pale yellow color, respectively. The hydrogen bonds are shown as black dotted line between tubulin residues and DAMA-colchicine, (A) Hydrogen bonding between DAMA-colchicine (green color) and tubulin 1SA0 residues i.e. Cys-239(2.20Å), Lys-350(2.31Å) and Val-181(1.91Å) at the interface binding pocket. (B) Hydrogen bonding between DAMA-colchicine and αβII tubulin isotype residues i.e. Lys-350(2.07Å), Cys-239(2.05Å) and Asn-256(2.15Å) at binding pocket. (C) Hydrogen bonding interaction between with DAMA-colchicine and αβIII tubulin isotype residues i.e. Lys-350(2.65Å) of β-tubulin and Asn-101(2.20Å) α-tubulin. (D) Hydrogen bonding between DAMA-colchicine and αβIV tubulin isotype i.e. Cys-239(2.08Å) and Lys-352(2.25Å) in the binding pocket. (TIF) [file pone.0156048.s005.tif]

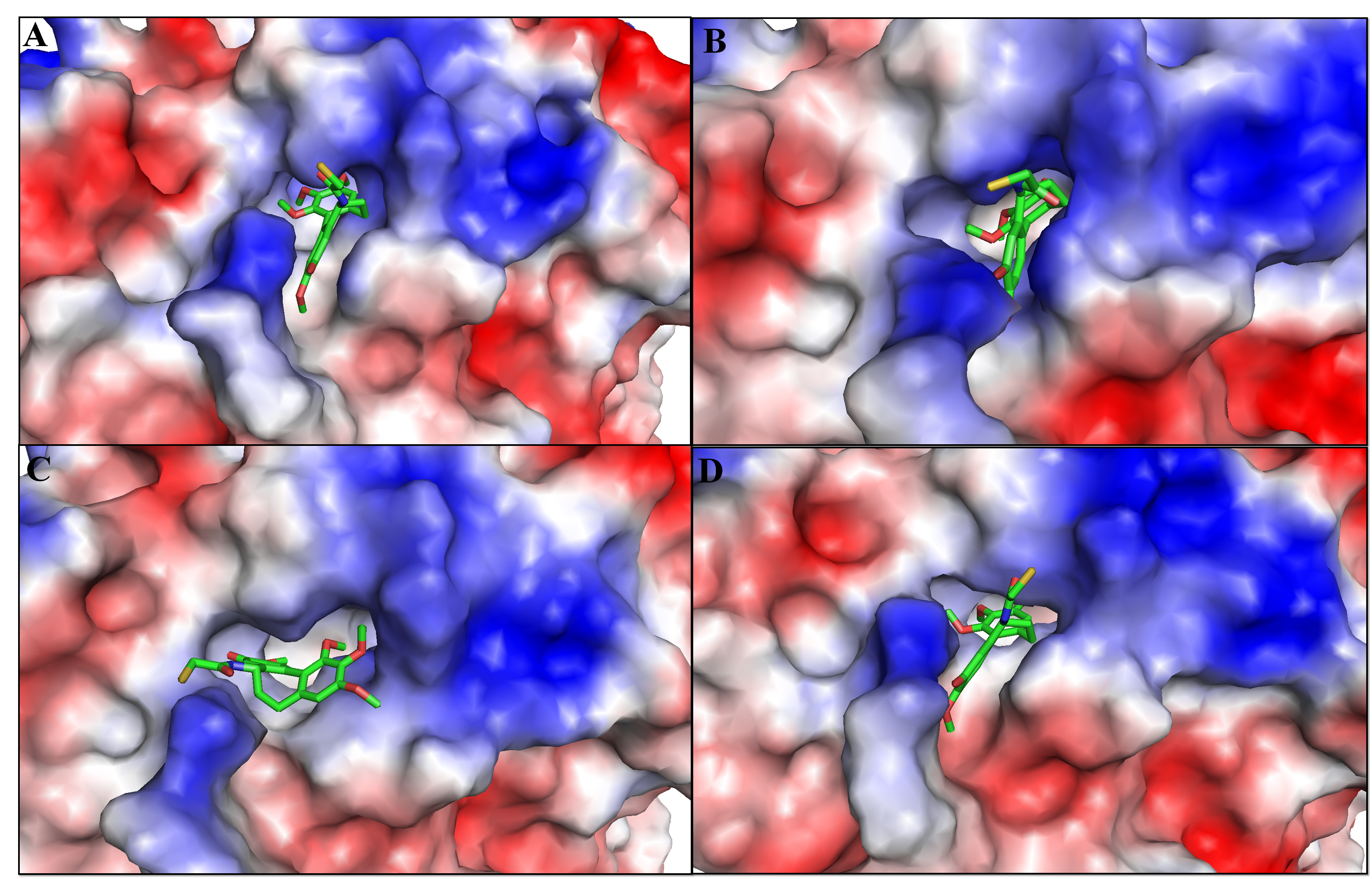

Supplement: S6 Fig — The red, blue and white color represents the negative, positive and neutral electrostatic potentials, respectively. The drug DAMA-colchicine bind at the interface of cavity of β-tubulin in tubulin 1SA0 and tubulin isotypes. DAMA-colchicine is shown in green color;oxygen, nitrogen, and sulphur atoms are shown in red, blue, and golden yellow colors respectively. (A) β-tubulin 1SA0 and DAMA-colchicine complex (B) βII tubulin isotype and DAMA-colchicine complex, (C) βIII tubulin isotype and DAMA-colchicine complex (D) βIV tubulin isotype and DAMA-colchicine complex. In βIII tubulin isotype, DAMA-colchicine prefers different conformation. (TIF) [file pone.0156048.s006.tif]

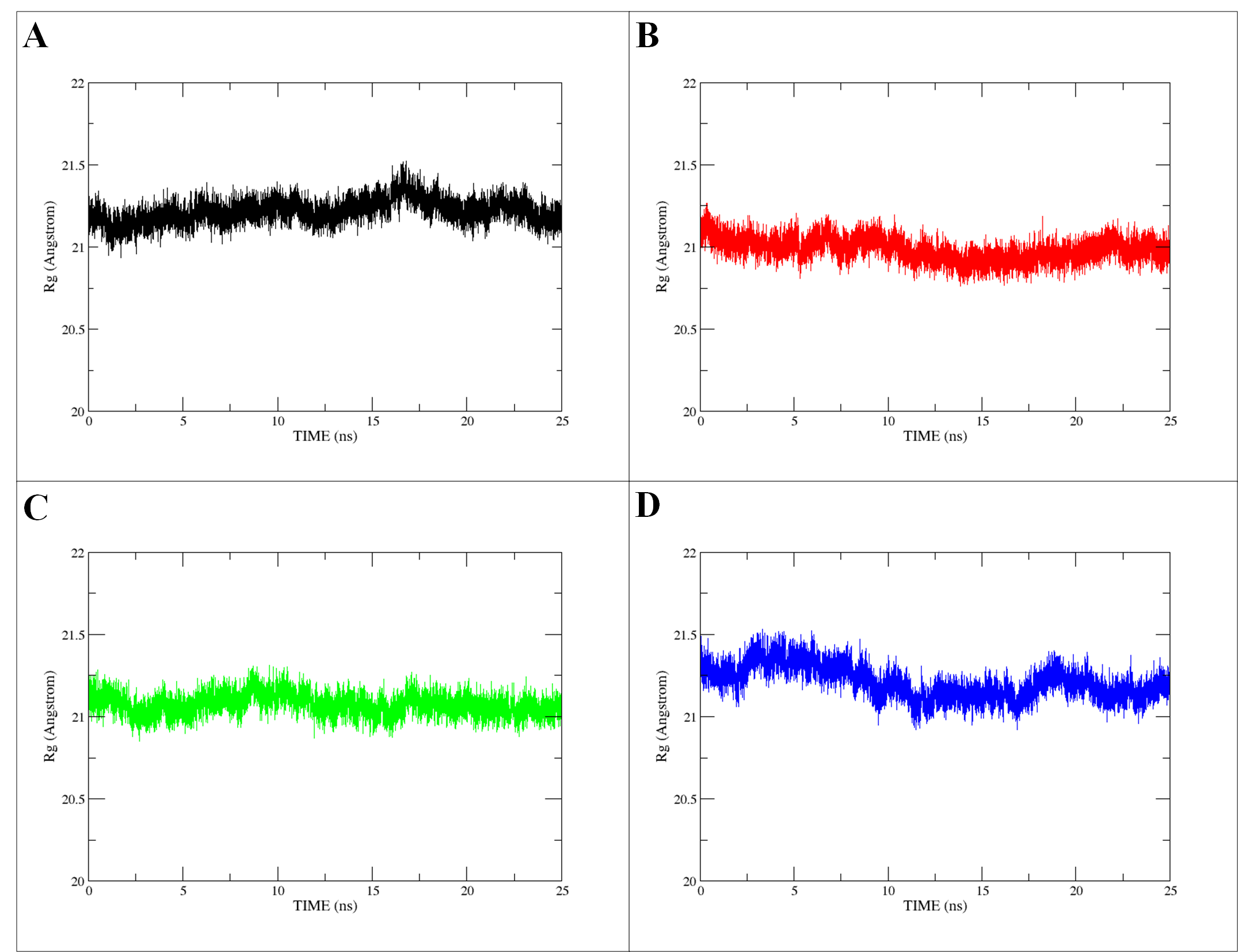

Supplement: S7 Fig — Radius of gyration (Rg) values correspond to (A) tubulin 1SA0 (black colour), (B) βII (red colour), (C) βIII (green colour) and (D) βIV (blue colour) of tubulin for 25ns MD simulations. The radius of gyration shows the compactness and stable behavior of β-tubulin structures, during molecular dynamics simulations, for tubulin 1SA0 and three β-tubulin isotypes. (TIF) [file pone.0156048.s007.tif]

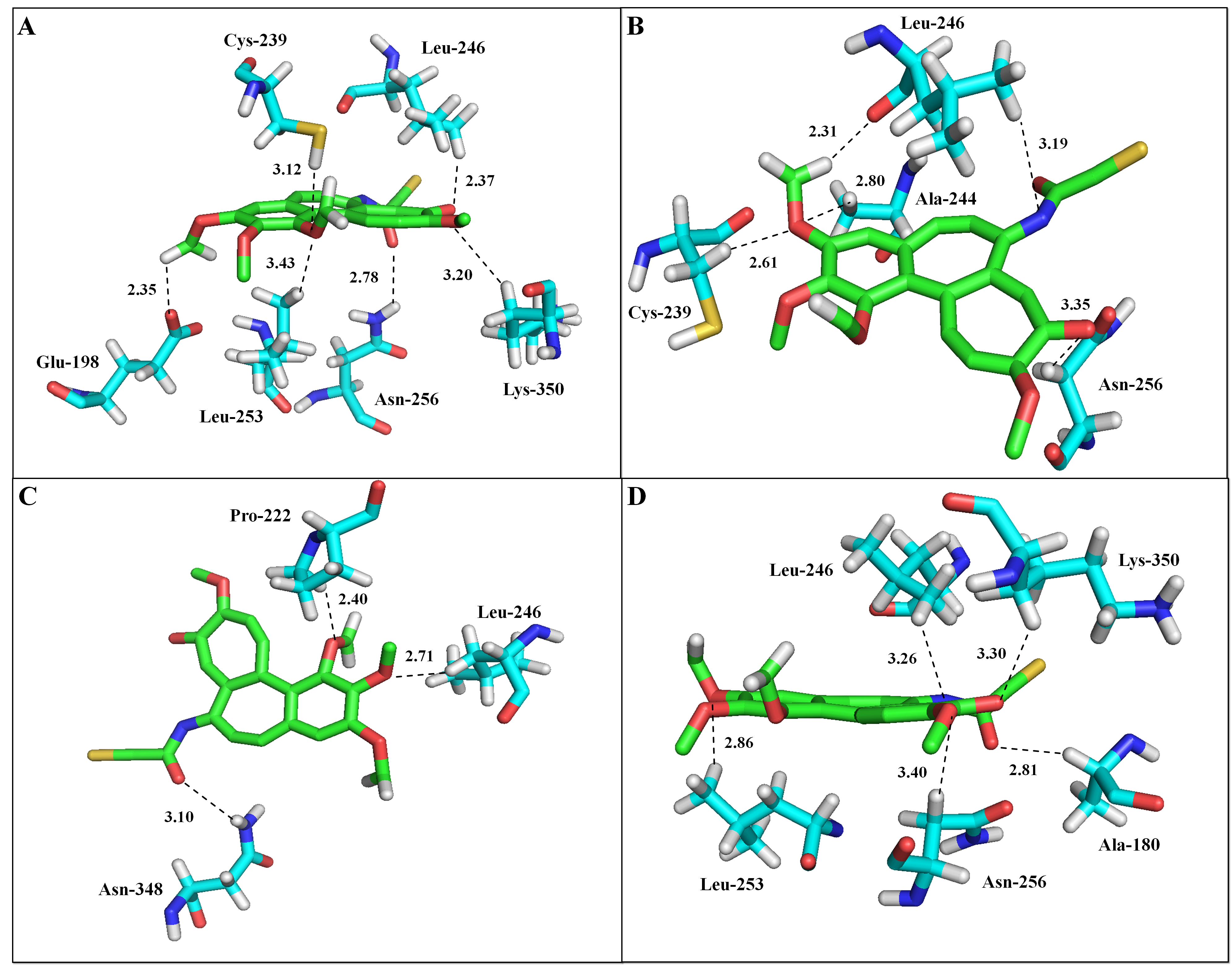

Supplement: S8 Fig — The residues involved in bonding have been shown in stick with cyan color. Hydrogen bonding between DAMA-colchicine and tubulin residues is shown with black dotted line. Crystal structure of DAMA-colchicine after molecular dynamics simulation is shown in green color. The oxygen, nitrogen and sulphur atoms of DAMA-colchicine has are shown in red, blue and pale yellow color, respectively. (A) Hydrogen bonding between DAMA-colchicine and tubulin 1SA0 amino acids i.e. Glu-198(2.35Å), Cys-239 (3.12Å), Leu-253 (3.43Å), Asn-256 (2.78Å) and Lys-350 (3.20Å) at binding pocket. (B) Hydrogen bonding between DAMA-colchicine and αβII tubulin isotype amino acids i.e. Cys-239(2.61Å), Leu-246 (3.19Å), Leu-246 (2.31Å) Asn-258 (3.35Å) and Ala-244 (2.80Å). (C) Hydrogen bonding between DAMA-colchicine and αβIII isotype amino acids i.e. Leu-246(2.71 Å) of β tubulin and Asn-348 (2.78Å) and Pro-222 (2.40Å) of α tubulin in binding pocket. (D) Hydrogen bonding interactions between DAMA-colchicine and αβIV isotype residues i.e. Leu-246 (3.26Å), Leu-253 (2.86Å), Asn-256 (3.40Å) and Lys-350 (3.30Å) of β chain and Ala-180(2.81Å) of α chain. (TIF) [file pone.0156048.s008.tif]

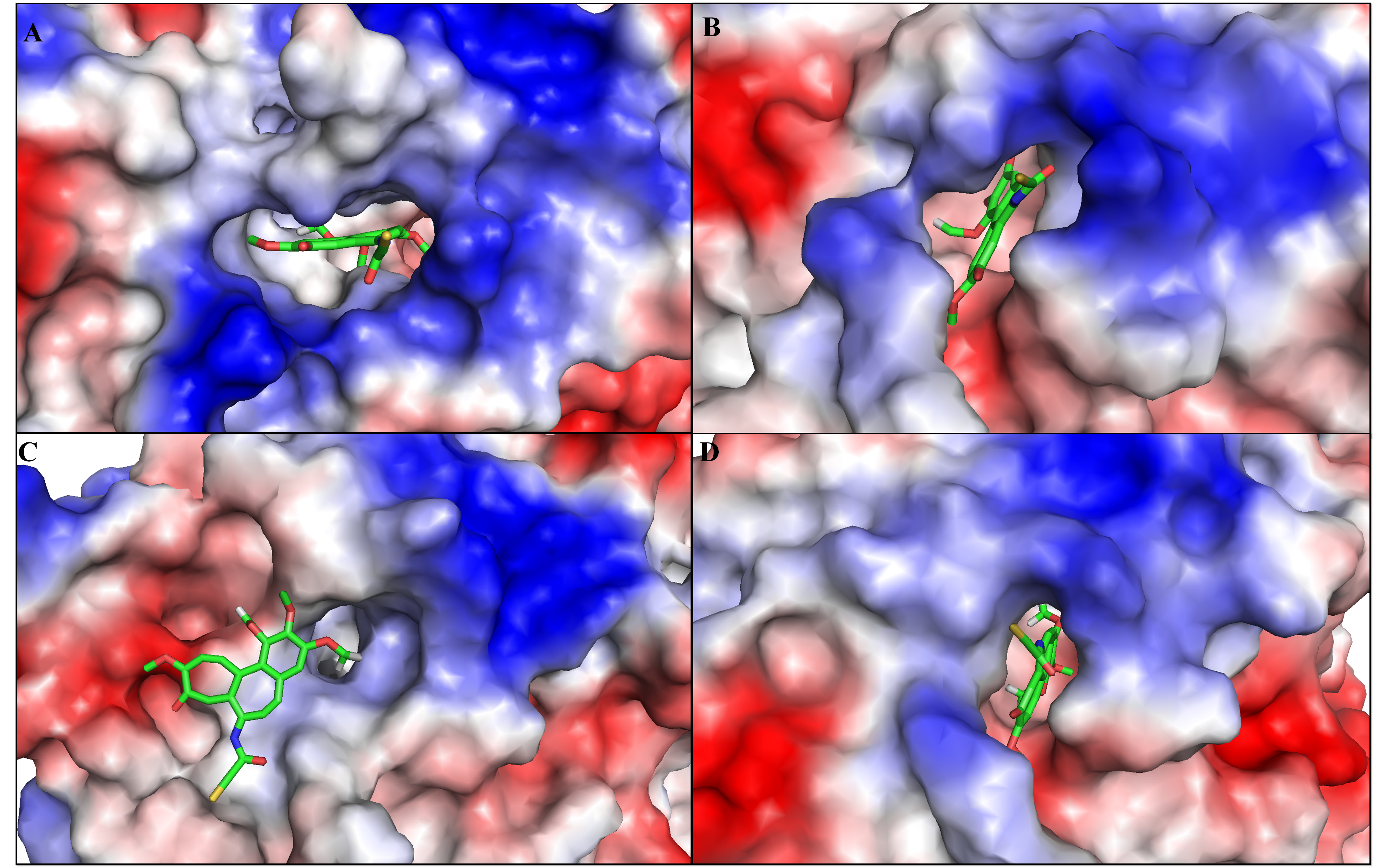

Supplement: S9 Fig — Colour scheme is same as shown in S6 Fig. (A) β-tubulin 1SA0 and DAMA-colchicine (B) βII tubulin isotype and DAMA-colchicine C) βIII tubulin isotype and colchincine and (D) βIV tubulin isotype and DAMA-colchicine. After MD simulation, the drug DAMA-colchicine located inside the binding pocket of β-tubulin in tubulin 1SA0 (A), βII (B) and βIV (D) isotypes whereas in βIII isotypes (C), it expelled from the binding pocket. (TIF) [file pone.0156048.s009.tif]

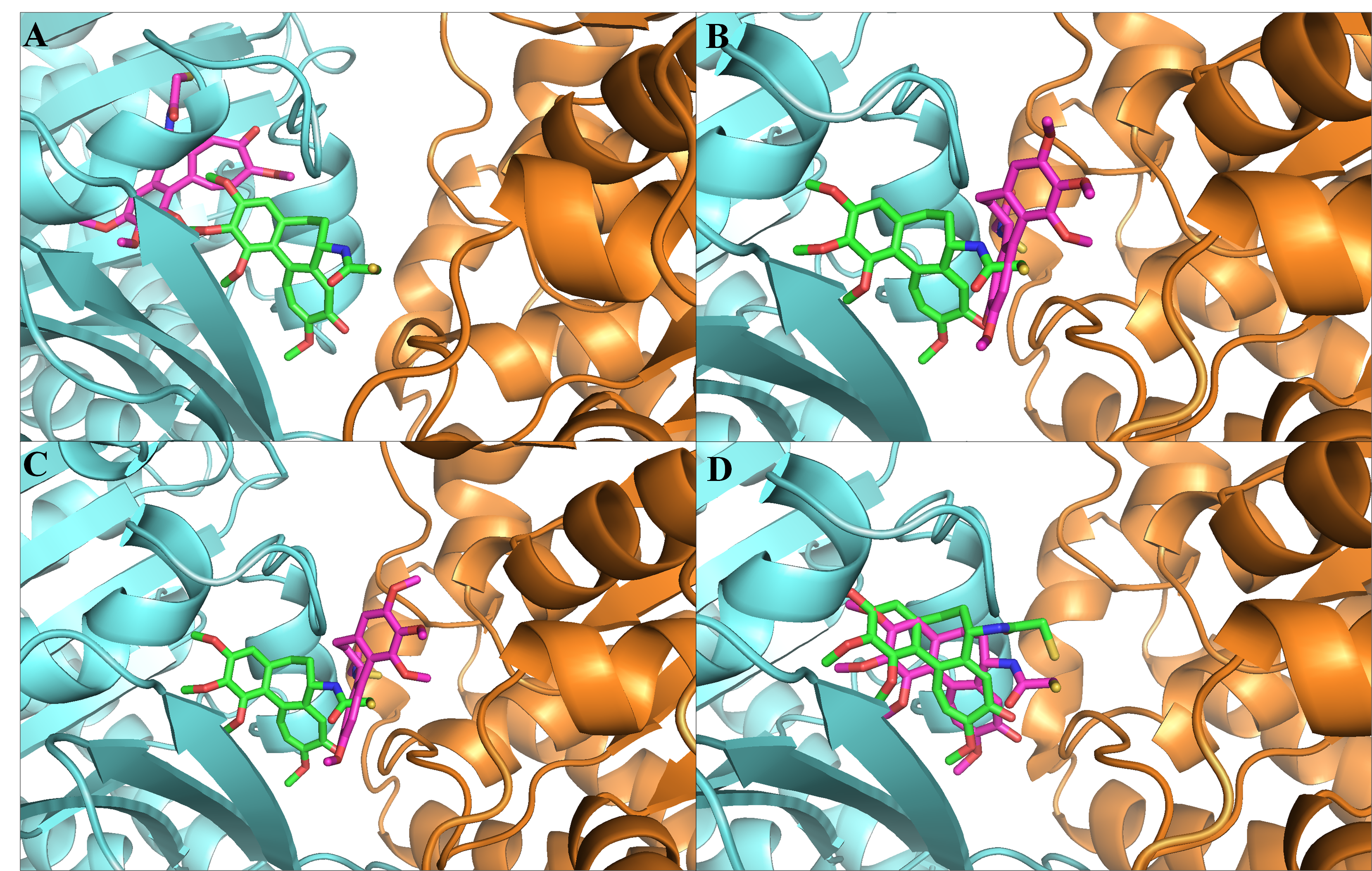

Supplement: S10 Fig — Color scheme for α-tubulin is tv_orange and β-tubulin is aquamarine. The conformation of DAMA-colchicine (shown in magenta) after docking with in-silico mutant structures of αβIII tubulin isotype (A) αβIII isotype with Ser 239 to Cys mutation, (B) αβIII isotype with Thr 315 to Ala, (C) αβIII isotype with Val 351 to Thr, (D) αβIII isotype with Ser 239-Cys, Thr315-Ala and Val351-Thr. The binding pose of DAMA-colchicine in crystal structure is shown in green. (TIF) [file pone.0156048.s010.tif]
