# Supplementary material for: Exploring the Origin of Differential Binding Affinities of Human Tubulin Isotypes αβII, αβIII and αβIV for DAMA-Colchicine Using Homology Modelling, Molecular Docking and Molecular Dynamics Simulations
Source: PLoS One. 2016 May 26;11(5):e0156048. doi: 10.1371/journal.pone.0156048 (PMC4882049; doi:10.1371/journal.pone.0156048)
Supplement: S1 Text — (A) Quality of Homology models of tubulin isotypes. (B) Experimentally measured binding energies of colchicine for bovine αβII αβIII and αβIV tubulin isotypes. (C) Experimentally measured binding energies of Desacetamidocolchicine (DAAC), a fast binding analogue of colchicine for bovine αβII αβIII and αβIV tubulin isotypes. (D) Molecular modeling and docking study of in-silico mutant structures of αβIII tubulin isotypes and DAMA-colchicine. (PDF) [file pone.0156048.s015.pdf]

S1 Text

(A) Quality of Homology models of tubulin isotypes:

The quality of template structure tubulin 1SA0 [1] (Fig 1) was evaluated by the online programs PROCHECK [2], VERIFY-3D [3] and ERRAT [4]. The PROCHECK checks the stereochemical quality of a protein structure. The input to PROCHECK is a single file containing the coordinates of protein structure and Ramachandran Plot is one of the outputs produced by PROCHECK. The **Ramachandran plot** produced by PROCHECK shows the **phi-psi** torsion angles for all residues in the structure (except those at the chain termini). Glycine residues are separately identified by triangles as these are not restricted to the regions of the plot appropriate to the other sidechain types. The different regions of the Ramachandran Plot are represented by different coloring/shading as described by Morris et al. [5]. The two "**most favoured regions"** are the **"core"** and **"allowed"** regions which correspond to **10°** x **10°** pixels having more than **100** and **8** residues in them, respectively. The **"generous"** regions were defined by Morris et al. [5] by extending out by **20°** (two pixels) all round the **"allowed"** regions. The space left after these regions have been defined as **"disallowed"** region. The **darkest** areas (shown in *red*) correspond to the **"core"** regions representing the most **favourable** combinations of **phi-psi** values. The higher percentage of residues in the **"core"** regions is one of the better guides to stereochemical quality. The PROCHECK result shows that 76.5% residues are in favored regions, 18% residues in additional allowed regions, 4.1% of residues in generously allowed regions and 1.3% residues in disallowed regions (Figure A in S1 Fig). The VERIFY-3D score was 97.86 (Figure B in S1 Fig), and the ERRAT score was 77.25 (Figure C in S1 Fig), which further indicated the good quality of the template model (Tubulin 1SA0.pdb). Thus, the quality of template model was very

good for homology modeling of $\alpha\beta_{II}$, $\alpha\beta_{III}$ and $\alpha\beta_{IV}$ tubulin heterodimers. The quality of these different αβ-tubulin isotypes model were also determined using the PROCHECK, VERIFY-3D and ERRAT score for $\alpha\beta_{II}$, $\alpha\beta_{III}$ and $\alpha\beta_{IV}$ tubulin isotype (S2-S4 Figs). The PROCHECK results also show the good quality of models (Figure A in S2-S4 Figs). The VERIFY-3D scores for $\alpha\beta_{II}$, $\alpha\beta_{III}$ and $\alpha\beta_{IV}$ were 95.81%, 95.25%, 94.99% (Figure B in S2-S4 Figs) and ERRAT scores were 88.05, 88.60 and 89.84 respectively (Figure C in S2-S4 Figs). The RMSD of template structure (wild type) and $\alpha\beta_{II}$, $\alpha\beta_{III}$ and $\alpha\beta_{IV}$ tubulin isotype heterodimer calculated using Chimera [6], the RMSD values for $\alpha\beta_{II}$, $\alpha\beta_{III}$ and $\alpha\beta_{IV}$ tubulin are 0.437Å, 0.468Å, 0.448Å respectively.

(B) Experimentally measured binding energies of colchicine for bovine $\alpha\beta_{II}$ $\alpha\beta_{III}$ and $\alpha\beta_{IV}$ tubulin isotypes.

**Protein System**	**Measured Affinity Constant, Ka [10] (M^{-1})**	**Calculated Binding Energy from Ka (kcal/mol)**
**$\alpha\beta_{II}$ isotype**	0.24×10^6	-7.39
**$\alpha\beta_{III}$ isotype**	0.12×10^6	-6.97
**$\alpha\beta_{IV}$ isotype**	3.31×10^6	-8.95

(C) Experimentally measured binding energies of Desacetamidocolchicine (DAAC), a fast binding analogue of colchicine for bovine $\alpha\beta_{II}$ $\alpha\beta_{III}$ and $\alpha\beta_{IV}$ tubulin isotypes.

Protein System	Measured Affinity Constant, Ka[11] (M^{-1})	Calculated Binding Energy from Ka (kcal/mol)
$\alpha\beta_{II}$ isotype	2.5×10^{6}	-8.78
$\alpha\beta_{III}$ isotype	1.5×10^{6}	-8.48
$\alpha\beta_{IV}$ isotype	4.0×10^{6}	-9.06

(D) Molecular modeling and docking study of in-silico mutant structures of $\alpha\beta_{III}$ tubulin isotypes and DAMA-colchicine:

The homology model of $\alpha\beta_{III}$ tubulin isotype was used to build the four different in-silico mutant structures of $\alpha\beta_{III}$ tubulin isotypes using the Swiss PDB Viewer program [7]: (1) $\alpha\beta_{III}$ isotype with Ser239 to Cys (2) $\alpha\beta_{III}$ isotype with Thr315 to Ala (3) $\alpha\beta_{III}$ isotype Val351 to Thr and (4) $\alpha\beta_{III}$ isotype with Ser239-Cys, Thr315-Val and Val 351-Thr. The generated models were used for the molecular docking of DAMA-colchicine to pinpoint the effect of individual residue changes on the binding affinity of DAMA-colchicine to $\alpha\beta_{III}$ isotypes The protocol for molecular docking using AutoDock4.2 [8] is same as discussed in “Molecular Docking of DAMA-colchicine and αβ Tubulin Isotypes” of Material and Methods section. The figures are generated using PyMol [9].

S1 Text References

1. Ravelli RBG, Gigant B, Curmi PA, Jourdain I, Lachkar S, Sobel A, et al. Insight into tubulin regulation from a complex with colchicine and a stathmin-like domain. Nature. 2004;428: 198–202.
2. Laskowski RA, MacArthur MW, Moss DS, Thornton JM. PROCHECK: a program to check the stereochemical quality of protein structures. Journal of Applied Crystallography. 1993. pp. 283–291.
3. Bowie JU, Lüthy R, Eisenberg D. A method to identify protein sequences that fold into a known three-dimensional structure. Science. 1991;253: 164–170.
4. Colovos C, Yeates TO. Verification of protein structures: patterns of nonbonded atomic interactions. Protein Sci. 1993;2: 1511–1519.
5. Morris a. L, MacArthur MW, Hutchinson EG, Thornton JM. Stereochemical quality of protein structure coordinates. Proteins Struct Funct Genet. 1992;12: 345–364.
6. Pettersen EF, Goddard TD, Huang CC, Couch GS, Greenblatt DM, Meng EC, et al. UCSF Chimera--a visualization system for exploratory research and analysis. J Comput Chem. 2004;25: 1605–1612.
7. Johansson MU, Zoete V, Michielin O, Guex N. Defining and searching for structural motifs using DeepView/Swiss-PdbViewer. BMC Bioinformatics. 2012. p. 173.
8. Morris GM, Ruth H, Lindstrom W, Sanner MF, Belew RK, Goodsell DS, et al. Software news and updates AutoDock4 and AutoDockTools4: Automated docking with selective receptor flexibility. J Comput Chem. 2009;30: 2785–2791.
9. DeLano WL. The PyMOL Molecular Graphics System, Version 1.1. Schrödinger LLC. 2002; http://www.pymol.org.
10. Banerjee A, Luduena RF. Kinetics of colchicine binding to purified beta-tubulin isotypes from bovine brain. J Biol Chem. 1992;267: 13335–13339.
11. Banerjee a, D'Hoore a, Engelborghs Y. Interaction of desacetamidocolchicine, a fast binding analogue of colchicine with isotypically pure tubulin dimers alpha beta II, alpha beta III, and alpha beta IV. J Biol Chem. 1994;269: 10324–9.
